# Effects of Time-Varying Parent Input on Children’s Language Outcomes Differ for Vocabulary and Syntax

**DOI:** 10.1177/0956797620970559

**Published:** 2021-03-15

**Authors:** Catriona Silvey, Özlem Ece Demir-Lira, Susan Goldin-Meadow, Stephen W. Raudenbush

**Affiliations:** 1 Division of Psychology and Language Sciences, University College London; 2 Department of Psychological and Brain Sciences, The University of Iowa; 3 DeLTA Center, The University of Iowa; 4 Iowa Neuroscience Institute, The University of Iowa; 5 Department of Psychology, The University of Chicago; 6 Department of Comparative Human Development, The University of Chicago; 7 Department of Sociology, The University of Chicago

**Keywords:** language development, environmental effects, causal analysis

## Abstract

Early linguistic input is a powerful predictor of children’s language outcomes. We investigated two novel questions about this relationship: Does the impact of language input vary over time, and does the impact of time-varying language input on child outcomes differ for vocabulary and for syntax? Using methods from epidemiology to account for baseline and time-varying confounding, we predicted 64 children’s outcomes on standardized tests of vocabulary and syntax in kindergarten from their parents’ vocabulary and syntax input when the children were 14 and 30 months old. For vocabulary, children whose parents provided diverse input earlier as well as later in development were predicted to have the highest outcomes. For syntax, children whose parents’ input substantially increased in syntactic complexity over time were predicted to have the highest outcomes. The optimal sequence of parents’ linguistic input for supporting children’s language acquisition thus varies for vocabulary and for syntax.

One of the most broadly studied topics in child development is the association between parents’ language input and children’s language development. Previous literature shows that variation in the quantity and quality of the language children receive from caregivers robustly predicts children’s language outcomes (e.g., Cartmill et al., 2013; Hart & Risley, 1995; Hoff, 2003; Huttenlocher et al., 1991, 2002; Rowe et al., 2009; Rowe & Goldin-Meadow, 2009; Weizman & Snow, 2001). However, two important questions about this relation remain unanswered: Does the impact of parent language input (a) vary over developmental time and (b) vary with the specific child language skill measured? Our goal in this research was to address these questions by examining longitudinal, dynamic relations between parent language input and two child language outcomes: vocabulary and syntax (the structured arrangement of words). We use novel statistical techniques to answer these time-dependent questions in a way that was not previously possible.

Our first question concerns the timing of the association between parent input and child outcomes. Researchers typically record interactions between the primary caregiver and child at a single time point, often before the child begins to utter words; they later measure the child’s language outcomes (Hoff, 2003; Huttenlocher et al., 2002; Weizman & Snow, 2001). In these paradigms, parent input measured early reliably predicts children’s later outcomes in both vocabulary (Hart & Risley, 1995; Huttenlocher et al., 1991) and syntax (Gleitman et al., 1984; Huttenlocher et al., 2002; Rowe et al., 2009) after analyses control for relevant parent and child background characteristics. However, because most studies track parent input at only one time point, the extent to which early input is key is not clear. Early language input could set in motion a growth process that leads to high levels of language development; early exposure to a wide range of sounds, meanings, and structures would then be sufficient for subsequent child language development, and later input would be less important. Although consistent with this *sensitive-period hypothesis* (Newport, 2006), the data collected thus far are also consistent with other explanations. One is that parents who provide high input early are likely to continue to do so throughout development; later parent input (and not early input) might then be what actually triggers child language growth. Another possibility is that the impacts of early input and later input combine; in other words, it is not parent input at one point but rather sequences of early and late input that best predict child outcomes. To adjudicate among these explanations, we need to examine parent–child interactions over time and compare sequences of parent input as predictors of later child outcomes.

The second question concerns the specificity of the association between parent input and child outcomes. Vocabulary and syntax have related, yet distinct, developmental trajectories. Thus, we might expect that the time in development when parent input has its largest impact on child outcome will vary for these two skills. Children start producing their first words around their first birthday, and their vocabulary continues to increase in size and diversity throughout preschool. Children start combining words into sentences between 1.5 and 2 years of age, produce basic syntactic operations such as negation between 2 and 3 years, and diversify their syntactic forms throughout preschool (Hoff, 2013). Parent input may then have an impact on vocabulary earlier in children’s development than it does on syntax. However, children might benefit from receiving syntactically rich input even before they start producing complex syntax themselves (Gomez & Gerken, 1999; Hirsh-Pasek & Golinkoff, 1999; Naigles, 1990). Early parent input could then have an impact not only on vocabulary development but also on syntactic development.

The majority of previous work has focused on the role of parent input in vocabulary development. Much less is known about its role in syntactic development, and even less is known about the effects of input on vocabulary and syntactic development in the same child. One exception is a study that examined parent input in relation to both vocabulary and syntax in the same children (Rowe et al., 2009); however, the study focused on input at one time point, and thus the researchers could not ask whether the period during which parent input has its biggest effect varies for vocabulary and syntax.

**Statement of Relevance**

The way parents talk to their children has a crucial impact on the development of children’s language skills. Previous work has shown that the number of unique words parents use early in their child’s development predicts the child’s later vocabulary. However, the impact of parents’ language input on children’s syntax—the grammatical combination of words—is less clear. Furthermore, analysis techniques used in previous research cannot discriminate between effects of earlier and later input. We applied novel statistical techniques to examine the effects of earlier and later language input on children’s vocabulary and syntax. For vocabulary, children whose parents used many unique words both earlier as well as later in their development had the best outcomes. For syntax, children whose parents’ input increased in syntactic complexity over time had the best outcomes. This work has implications for parents and caregivers seeking to optimally support children’s language development.

Our goal was to compare, for the first time, the impact that the timing of parent input has on child vocabulary and syntax development. We measured parent vocabulary and syntax input earlier (age 14 months) and later (age 30 months) in a child’s development, and we tested three hypotheses with respect to child vocabulary and syntactic outcomes: (a) Earlier parent input is more important than later input, (b) later parent input is more important than earlier input, and (c) the sequencing of parent input is key.

As in previous studies, we controlled for baseline covariates, such as household income. However, examining input at multiple time points introduces another type of confounding. Parent speech is part of a dynamic process in which early parent input shapes intermediate child outcomes, which, in turn, shape later input and later outcomes (Irvin et al., 2016). Parents are likely to adjust their later input to the response the child gave to earlier input. A response to early input that predicts later input and long-term outcome is called a *time-varying confounder*. These confounders prevented us from using standard methods to assess the true impact of parent input on child outcome. We used methods derived from epidemiology (Naimi et al., 2014; Robins et al., 2000) to control for time-varying confounders, as well as time-invariant baseline covariates, and thus estimate the true effect of earlier and later parent input on child language outcomes measured in kindergarten, under the assumption that our observed covariates adequately captured confounding.

We thus attempted to provide novel answers to one of the longest-standing questions in developmental psychology—the role of parents’ language input in children’s language development. In so doing, we also tackled a central methodological challenge to exploring broader questions regarding the mechanisms that underlie the intergenerational transmission of cognitive skill.

## Method

Data were taken from a longitudinal study of language development (Goldin-Meadow et al., 2014), which was approved by The University of Chicago Institutional Review Board. Participants were 64 children (31 girls; 36 firstborn children) and their primary caregivers, selected to be representative of the greater Chicago area in terms of race, ethnicity, and income. This sample size is larger than in any existing longitudinal study of child language development with comparable observation intervals. Of the primary caregivers, 56 were mothers and one was a father; in the remaining seven families, mother and father were joint primary caregivers. Children were visited at home every 4 months from age 14 months to age 58 months and were videotaped for 90 min engaging in typical interaction with their caregivers. All speech and gestures by the primary caregiver and the child were transcribed. From these transcripts, we calculated measures of baseline and time-varying child language and measures of parent vocabulary and syntax input.

### Time points

Our research questions concern the effects of parent language input given earlier and later during child language development. We therefore needed to select time points for “earlier” and “later” on a principled basis so that the time periods related to distinct stages in a typically developing child’s learning trajectory. Earlier input should be measured at a time when the child’s own language production is still very limited. Later input should be measured during a qualitatively different period when the child’s language is beginning to become more sophisticated. At 14 months, most children are just beginning to produce their first words: The median number of unique word types produced by children in our sample during the 14-month observation session was 8.5. We therefore chose 14 months as our time point for earlier input. Thirty months is the median session in which children in our sample began to produce utterances that contained more than one clause, indicating that, by 30 months, the children’s language was becoming more complex (cf. Vasilyeva et al., 2008). We therefore chose 30 months as our time point for later input.

We chose to measure child vocabulary and syntax outcomes in kindergarten for two reasons: (a) Kindergarten typically marks the beginning of the period during which children receive oral and written language input in formal schooling contexts, and (b) children’s receptive language skills measured at school entry are a significant predictor of future academic achievement (Duncan et al., 2007; Pagani et al., 2010).

### Variables

#### Nonlinguistic covariates

We used annual household income and primary caregiver years of education as covariates in our analyses. Income was coded as one of six categories: between $0 and $14,999, between $15,000 and $34,999, between $35,000 and $49,999, between $50,000 and $74,999, between $75,000 and $99,999, and $100,000 or above. The midpoint of each category in thousands of dollars was assigned as the value of household income, except for the highest category, which was assigned a value of 100. Education was coded as one of five categories: some high school (10 years), high school/GED (12 years), some college or trade school (14 years), bachelor’s degree (16 years), and advanced degree (18 years). Income and education were collected by parent report at each visit. We measured income and education as baseline covariates, taking the values reported at the first visit when the children were 14 months old. For 36 families, the income category did not change during the course of the study. Of the remaining 28 families, 23 ended the study within one income category from where they had started, four were two categories higher, and one was three categories higher. Years of education did not change during the course of the study for any caregiver. For families with joint primary caregivers, we took the mother’s years of education. For three of the seven joint caregivers, the mother’s years of education were identical to the father’s; for the remaining four, the mother’s and father’s education levels were within one category of each other.

To account for genetic similarities between parents and children, as well as other parental influences, we measured the verbal IQ of the primary caregiver when children were in fifth grade using the vocabulary *t* score from the second edition of the Wechsler Abbreviated Scale of Intelligence (WASI; Wechsler, 2011). Where mother and father were joint primary caregivers, only the mother’s verbal IQ was measured, with one exception (a joint caregiver family in which only the father elected to take the WASI). Our remaining nonlinguistic covariates were child gender and child birth order. Following previous research on the influence of birth order on language development (e.g., Hoff-Ginsberg, 1998), we coded birth order as one of two categories: (a) firstborn or only child and (b) second or later-born child. There might be further differences between second-born and later-born children (Kristensen & Bjerkedal, 2007), but in our small sample, only 11 children had two or more siblings; of these 11, only three had three or more siblings. Further, the categorical and continuous measures of birth order were significantly correlated, *r* = .78, 95% confidence interval (CI) = [.66, .86], *p* < .01.

#### Baseline measures of child language and gesture

From transcripts of the 14-month visit, we calculated child word types, defined as the number of unique words the child produced during the observation session, and used this as a baseline measure of the child’s productive vocabulary, which could influence both child outcomes and later parent input. We also calculated child gesture types, defined as the number of unique meanings the child conveyed in gesture. Previous work found that early gesture is associated with later vocabulary and syntax (Rowe & Goldin-Meadow, 2009), motivating us to include this measure as one of our covariates.

#### Measure of parent vocabulary input

From transcripts of the 14-month (earlier input) and 30-month (later input) visits, we calculated parent word types, defined as the number of unique words the parent produced during the observation session. We followed many previous studies (e.g., Bornstein et al., 1998; Hoff & Naigles, 2002) in using word types to measure the diversity of the vocabulary to which the child is exposed. Word tokens, or the total number of words addressed to the child, are also important in fostering child vocabulary development (Huttenlocher et al., 1991). However, in most samples, including ours, word types and tokens are highly correlated (*r* = .89, 95% CI = [.82, .93], at 14 months, *r* = .91, 95% CI = [.85, .94], at 30 months); as a result, their potential independent contributions to child vocabulary cannot be easily disentangled.

#### Measure of parent syntax input

From transcripts of the 14-month visit (earlier input) and 30-month visit (later input), we calculated the syntactic complexity of parent speech, defined as the number of clauses per sentence that the parent produced during the observation session. Following Huttenlocher et al. (2002), we excluded utterances that were not complete sentences (i.e., utterances not containing a verb) from the calculation. We chose number of clauses per sentence, rather than absolute number of multiclause sentences, because Huttenlocher et al. showed that the absolute number of multiclause sentences in parent input did not predict child language complexity. In our data, we also found that the absolute number of multiclause sentences was highly correlated with the total number of utterances the parent produced (.65 < *r* < .78). Huttenlocher et al. used the proportion of multiclause parent sentences as their predictor; we chose instead to use number of clauses per sentence, as this gave us a more fine-grained, and more statistically robust, measure of syntactic complexity in the input. Finally, we multiplied this value by 100 to obtain the number of clauses per 100 sentences so that our measures of vocabulary and syntax input would be on similar scales.

#### Time-varying measure of child language

From tran-scripts of the 26-month visit, we calculated child word types as described earlier. We also calculated child mean length of utterance in words. In English, mean length of utterance in words correlates almost perfectly (*r* = .998) with mean length of utterance in morphemes (Parker & Brorson, 2005); mean length of utterance in words can also be calculated more reliably and requires fewer unwarranted theoretical commitments about the nature of children’s representations. We averaged children’s *z* scores on this syntactic measure with their *z* scores on word types to create a composite measure of child language during the period between earlier input (14 months) and later input (30 months).

#### Vocabulary outcome

For the child’s vocabulary outcome, we chose the third edition of the Peabody Picture Vocabulary Test (PPVT; Dunn & Dunn, 1997), a widely used assessment of receptive vocabulary. The PPVT was administered to children at several time points throughout the study. We were interested in children’s vocabulary skill in kindergarten. To increase the reliability of our PPVT estimate, we combined the administrations of PPVT when children were 42 months old, 54 months old, in preschool, in kindergarten, and in second grade into a growth model centered at 74 months, the median age at which our syntax outcome (which was administered only once) was measured. The child-specific intercept from this growth model represented our best estimate of the child’s true standardized PPVT score in kindergarten. Details of the growth model are reported in Section S1 in the Supplemental Material available online.

#### Syntax outcome

When the children were in kindergarten (median age = 74 months), they completed the Recalling Sentences subtest from the Clinical Evaluation of Language Fundamentals (CELF; Semel et al., 2003). In this test, the child is asked to repeat sentences of increasing length and complexity. Previous work suggests that sentence-repetition tasks are a valid index of children’s language skills (Klem et al., 2015), and the CELF is recognized as one of the more reliable and valid language-evaluation instruments available (Denman et al., 2017). We were unable to build growth models for CELF because it was administered only once. The children’s standardized scores on the CELF Recalling Sentences (CELF-RS) subtest constituted our syntax outcome. We used standardized scores to ensure comparability across children because their exact age when the CELF was administered varied.

Descriptive statistics for all variables are shown in Table 1. Note that our data set contains missing values. We addressed these omissions via multiple imputation with the method of predictive mean matching, implemented using the *mice* library in R (van Buuren & Groothuis-Oudshoorn, 2011). See Section S2 in Supplemental Material for details of the imputation procedure and an alternative analysis that used only complete cases.

**Table 1.** Descriptive Statistics for All Baseline Covariates *X*_0_, Time-Varying Covariate *X*_1_, Input Variables *Z*_1*V*_ and *Z*_2*V*_ for Vocabulary and *Z*_1*S*_ and *Z*_2*S*_ for Syntax, Vocabulary Outcome *Y_V_*, and Syntax Outcome *Y_S_*

Variable	Type	Number of valid cases	Min.	*M*	*Mdn*	Max.	*SD*
Child gesture types at 14 months	*X*_0_	64	4	21.70	18.5	54	12.49
Child word types at 14 months	*X*_0_	64	0	14.06	8.5	59	14.57
Parent verbal IQ	*X*_0_	51	37	57.88	57	80	10.66
Household income (thousands of dollars)	*X*_0_	64	7.5	60.20	62.5	100	31.42
Parent years of education	*X*_0_	64	10	15.66	16	18	2.24
Composite *z* score of child word types and mean length of utterance at 26 months	*X*_1_	61	−1.65	0	0.01	1.99	0.93
Parent word types when child was 14 months old	*Z*_1*V*_	64	62	403.72	407	720	125.18
Parent word types when child was 30 months old	*Z*_2*V*_	61	178	464.13	486	740	126.58
Cumulative parent word types	*Z*_1*V*_ + *Z*_2*V*_	61	402	870.26	880	1307	227.11
Parent clauses per 100 sentences when child was 14 months old	*Z*_1S_	64	100	110.27	110	122	4.19
Parent clauses per 100 sentences when child was 30 months old	*Z*_2S_	61	103	116.62	116	129	6.05
Child standardized PPVT score in kindergarten (intercept from growth model)	*Y*_V_	60	66.49	110.47	112.63	137.77	13.39
Child standardized CELF Recalling Sentences score in kindergarten	*Y*_S_	54	3	10.70	11	16	2.98

Note: PPVT = Peabody Picture Vocabulary Test; CELF = Clinical Evaluation of Language Fundamentals.

### Procedure

Our goal was to investigate the timing and specificity of the relationship between parent input and child outcomes in vocabulary and syntax. However, in order to make valid inferences, we had to account for baseline covariates that were associated with levels of parent input and also with child language outcomes (e.g., household income, parent verbal IQ). Furthermore, we had to account for child language measured between the earlier and later input periods, a time-varying confounder that could be influenced by earlier input and could in turn influence later input.

If we were running an experiment, we would have assigned children at random to sequences of input and observed their outcomes. We could not follow this procedure in an observational study, but we could use a statistical approach that allowed us to treat observational data as if they were from a randomized experiment—*inverse probability of treatment weighting* (IPTW; Robins et al., 2000). The concept is simple. We gave more weight to children who were unlikely (given their covariates) to receive the sequence of input they received at 14 and 30 months. Conversely, we gave less weight to children who were likely (given their covariates) to receive the sequence of input they received. If the measured covariates adequately accounted for confounding, the weighted data would then resemble data from an experiment in which sequences of input were assigned at random. We adapted the quantile-binning approach (Naimi et al., 2014), a version of IPTW that can be used to adjust for confounding in the case of continuous input, which characterized our data. A full description of the method is provided in Section S3 in the Supplemental Material. For evidence that this method, originally developed in the context of large samples, also produces robust estimates in small samples such as ours, see the simulations reported in Section S5 in the Supplemental Material.

We were interested in the effect on child language of any possible sequence of inputs (i.e., *Z*_1_, *Z*_2_, where *Z*_1_ = earlier input and *Z*_2_ = later input). The primary hypotheses we wanted to test related to the timing of language input and its effect on children’s vocabulary and syntax outcomes. Specifically, our candidate hypotheses were (a) that earlier parent input is more important than later input, (b) that later parent input is more important than earlier input, and (c) that cumulative parent input is key, with timing being of little importance. Note that the answers to these questions may differ for vocabulary and syntax. In order to answer these questions, we estimated the statistical model

(1) $$Y_{i}\;=\;\alpha \;+\;\delta_{1}Z_{1i}\;+\;\delta_{2}Z_{2i}\;+\;e_{i},$$

where $Y_{i}$ is the language outcome (vocabulary or syntax) for child *i*; δ_1_ is the impact of each additional unit of parent input *Z*_1*i*_ received when the child was 14 months old, holding constant parent input *Z*_2*i*_ received when the child was 30 months old; δ_2_ is the impact of each additional unit of parent input *Z*_2*i*_ received when the child was 30 months old, holding constant parent input *Z*_1*i*_ received at age 14 months; α is the model intercept; and *e_i_* is a random error assumed to be uncorrelated with *Z*_1_ and *Z*_2_.

Under the null hypothesis, δ_1_ = δ_2_ = δ $\delta_{1}=\delta_{2}=\delta$ , input at each age is equally important, and what matters is simply the cumulative input, that is, $Y_{i}\;=\;\alpha \;+\;\delta (Z_{1i}\;+\;Z_{2i})\;+\;e_{i}$ . Assuming input is positive at each age but δ_1_ is greater than δ_2_, earlier input is more important than later input. Sensitive-period theory (that input during an early window is necessary and sufficient for later growth; see Newport, 2006) is a strong version of this hypothesis, that is, δ_1_ > 0, δ_2_ = 0. A similarly strong hypothesis, δ1 = 0, = δ2 > 0, indicates that earlier input is unimportant and that later input is necessary and sufficient for growth.

As a preliminary step, we also estimated the apparent effect of earlier input without controlling for later input, an approach taken in many previous studies. To do this, we estimated the model

(2) $$Y_{i}\;=\;\alpha \;+\;\delta^{*}Z_{1i}\;+\;e_{i}.$$

Here, $\delta^{*}$ is the expected increment to the outcome Y associated with a unit increase in $Z_{1i}$ . We assumed here that the random error $e_{i}$ is uncorrelated with earlier input $Z_{1}$ after controlling for observed baseline covariates through weighting. Even when this assumption is true, ambiguity surrounds the interpretation of $\delta^{*}$ . On the one hand, $\delta^{*}$ is equal to δ_1_ only when δ_2_ is set to 0 (i.e., later input has no effect), corresponding to sensitive-period theory. On the other hand, if $\delta_{2}$ does not equal 0, then $\delta^{*}$ represents the joint effect of earlier and later input combined in some unspecified manner. The ambiguity of $\delta^{*}$ is one of the primary motivations for our study. Whereas the studies reviewed earlier focused on the association between earlier input and later outcomes, effectively seeking an inference about $\delta^{*}$ , our goal was to study $\delta_{1}$ and $\delta_{2}$ , the impacts of parent input at $Z_{1}$ and $Z_{2}$ Z2.

Finally, we wanted to assess the extent to which our results may be sensitive to two potential sources of bias. First, there may be confounders we did not observe. We needed to assess the magnitude of possible biases that could result from a range of potential unobserved confounders. Second, we assumed a linear relationship between parent input and child outcomes. We needed to assess the robustness of our results to differing assumptions about the functional form of the relationship between input and outcome. All analysis and simulation scripts are available at https://github.com/silveycat/vocab-syntax.

## Results

### Vocabulary

We divided the sample into eight quantiles by parent word types when children were 14 months old ( $Z_{1V}$ ) and used an ordinal model to predict quantile from covariates (see Section S4 in the Supplemental Material for simulations justifying our choice of eight quantiles, and see Section S6 for the procedure we used to assess covariates for inclusion). Covariates included in the model for $Z_{1V}$ were parent verbal IQ, household income, and child gender. We then generated weights for $Z_{1V}$ , as described in Section S3 in the Supplemental Material. Adjusting for these three covariates was sufficient to achieve balance on all baseline covariates. We then repeated the same process for parent word types when children were 30 months old ( $Z_{2V}$ ). Covariates included in this model were parent word types when the child was 14 months old ( $Z_{1V}$ ) and a composite measure of child language at 26 months ( $X_{1}$ ; see the “Time-varying measure of child language” subsection above). Adjusting for these two covariates was sufficient to achieve balance on all covariates. Balance checking and common support for the vocabulary models are reported in Tables S3 and S4 and Figures S3 and S4 in the Supplemental Material.

To replicate previous analyses that have assessed the impact of parent input on child outcomes, we first estimated Equation 2, which represents the effect of earlier input without accounting for later input. We found a ${\hat{\delta }}^{*}$ of 0.028, 95% CI = [0.004, 0.052], nominal *p* = .023. This effect corresponded to an expected 2.8 additional points on our vocabulary measure—standardized PPVT score in kindergarten—for every 100 additional word types the parent provided when the child was 14 months old. This result was in line with previous results but, as noted earlier, was ambiguous. This apparently strong effect could reflect the critical importance of earlier input, as emphasized in much of the past literature. However, the same result would arise if later input were critically important, simply because parents tend to provide stable vocabulary input; in our sample, the correlation between $Z_{1V}$ and $Z_{2V}$ for vocabulary was .60, 95% CI = [.41, .74].

To address this possibility, we estimated Equation 1, which represents the impact of possible sequences $\left(Z_{1i},Z_{2i}\right)$ of input using combined weights (see Section S3 in the Supplemental Material), so that we accounted for the child’s propensity to receive high input both earlier and later. The results can be seen in Table 2, Rows 1 and 2. We found a ${\hat{\delta }}_{1}$ of 0.013, 95% CI = [−0.014, 0.041], nominal *p* = .340, and a ${\hat{\delta }}_{2}$ of 0.029, 95% CI = [−0.000, 0.058], nominal *p* = .050. Under this model, the difference between the effects of earlier and later input was 0.017, 95% CI = [−0.031, 0.065] (not shown in Table 2). This CI included 0. We therefore retained the null hypothesis: $\delta_{1}\;=\;\delta_{2}\;=\;\delta ,\;p\;=\;.513$ (not shown in Table 2). Equation 1 reduces to $Y_{i}\;=\;\alpha \;+\;\delta (Z_{1i}\;+\;Z_{2i})\;+\;e_{i}$ , a constant-effects model in which earlier and later input make equal contributions to vocabulary. Our estimate ( $\hat{\delta }$ ) was 0.021, 95% CI = [0.007, 0.034], nominal *p* = .003 (Table 2, Row 3).

**Table 2.** Results of Weighted Outcome Models Estimating the Effect of Earlier (*Z*_1*V*_) and Later (*Z*_2*V*_) Parent Vocabulary Input on Children’s Standardized PPVT Scores in Kindergarten

Model	Predictor	Coefficient estimate ( $\hat{\delta }$ )	β	95% CI	*SE*	*t* ratio	Nominal *p*	AICc
Differing effects	*Z*_1*V*_	0.013	0.123	[−0.014, 0.041]	0.014	0.96	.340	496.4
Differing effects	*Z*_2*V*_	0.029	0.280	[−0.000, 0.058]	0.014	2.00	.050	496.4
Constant effects	*Z*_1*V*_ + *Z*_2*V*_	0.021	0.358	[0.007, 0.034]	0.007	3.06	.003	494.7
Earlier input is sufficient	*Z*_1*V*_	0.027	0.255	[0.004, 0.051]	0.012	2.30	.025	498.6
Later input is sufficient	*Z*_2*V*_	0.036	0.347	[0.011, 0.061]	0.012	2.91	.005	495.1

Note: Results are based on five imputed data sets, with estimates and standard errors pooled according to Rubin’s rules (Rubin, 1987). The mean Akaike information criterion adjusted for small sample size (AICc) is reported from the models run on the five imputed data sets. PPVT = Peabody Picture Vocabulary Test; CI = confidence interval.

Although the conclusion of equal contributions was parsimonious, our confidence in this conclusion was undermined by the weak power of the test of the null hypothesis $:\;\delta_{1}\;=\;\delta_{2}\;=\;\delta$ . Our estimate was 0.017 with a wide 95% CI of [−0.031, 0.065]. This CI does not rule out large differences between the effects of earlier and later input. The lack of power arises from the small sample size and the moderately high correlation between earlier and later vocabulary input.

Although we could not precisely estimate the difference between the impact of earlier and later input, we were able to test two strong models. The first was the model based on the sensitive-period theory, according to which $\delta_{1}$ was greater than 0 and $\delta_{2}$ was equal to 0. Estimation of this model (Table 2, Row 4) yielded the following results: ${\hat{\delta }}_{1}\;=\;0.027$ , 95% CI = [0.004, 0.051], nominal *p* = .025. An alternative strong model assumed that $\delta_{1}$ was equal to 0 and $\delta_{2}$ was greater than 0—that is, only later input matters. Estimation of this model (Table 2, Row 5) yielded the following results: ${\hat{\delta }}_{2}\;=\;0.036$ , 95% CI = [0.011, 0.061], nominal *p* = .005. Using the standard Akaike information criterion (AIC) method of model comparison (in which a lower AIC corresponds to a better model), we found that neither of these two strong models (Rows 4 and 5 of Table 2) fitted the data as well as the parsimonious model of equal contributions in which $\delta_{1}$ was equal to $\delta_{2}$ (Table 2, Row 3).

Our conclusion is that higher levels of input both earlier and later in development have a beneficial effect on vocabulary. There was no evidence in our data that earlier input is more important than later input in predicting vocabulary in kindergarten. Indeed, there was some suggestion that later input may be more important than earlier input, but our sample was too small to strongly warrant this claim. Our sensitivity analysis, reported in Section S8 in the Supplemental Material, suggested that reasonable levels of unobserved confounding would not lead to a bias of more than 24% of our estimate and would not qualitatively change our conclusions.

### Syntax

We followed the same procedure for syntax. We divided the sample into eight quantiles by parent clauses per 100 sentences at 14 months ( $Z_{1S}$ ) and used an ordinal model to predict quantile from covariates (see Section S6 in the Supplemental Material for the procedure we used to assess covariates for inclusion). A model for $Z_{1S}$ that included parent verbal IQ, child birth order, and child word types at 14 months achieved balance on all baseline covariates. A model for parent clauses per 100 sentences at 30 months ( $Z_{2S}$ ) that included parent clauses per 100 sentences at 14 months ( $Z_{1S}$ ), a composite measure of child language at 26 months ( $X_{1}$ ), parent verbal IQ, parent education, and child gender achieved balance on all covariates. Balance checking and common support for the syntax models are reported in Tables S5 and S6 and Figures S5 and S6 in the Supplemental Material.

To replicate what other researchers have done, we again first estimated Equation 2, which represents the effect of earlier input without accounting for later input. We found no clear effect of earlier input, ${\hat{\delta }}^{*}\;=\;-0.03$ , 95% CI = [−0.23, 0.18], nominal *p* = .792, on our syntax measure, the standardized CELF-RS score. This result is ambiguous. Given the width of the CI, we cannot rule out the possibility that high complexity in earlier input was truly associated with lower scores on our outcome measure and that later input may have a different effect. If this is the case, we may have a better chance of disentangling the effects of earlier and later input for syntax than we had for vocabulary, as the correlation between $Z_{1}$ and $Z_{2}$ for syntax (*r* = .37, 95% CI = [.13, .57]) was weaker than for vocabulary (*r* = .60, 95% CI = [.41, .74]).

To address this possibility, we estimated Equation 1, which represents the impact of possible sequences $\left(Z_{1i},Z_{2i}\right)$ of input using combined weights (see Section S3 in the Supplemental Material), so that we could account for the child’s propensity to receive high input both earlier and later. The results can be seen in Table 3. We found a ${\hat{\delta }}_{1}$ of −0.24, 95% CI = [−0.44, −0.03], nominal *p* = .024, and a ${\hat{\delta }}_{2}$ of 0.22, 95% CI = [0.05, 0.38], nominal *p* = .014. Under this model, we rejected the null hypothesis (earlier and later input have an equal and cumulative effect): $\delta_{1}\;=\;\delta_{2}\;=\;\delta ,\;p\;=\;.007$ (not shown in Table 3). Our data provide evidence that high syntactic complexity in later input has a more beneficial effect in promoting syntax skill than does high syntactic complexity in earlier input (when, at each time point, we control for input received at the other time point). This is shown by the 95% CI for our estimator of the difference between the effects of later and earlier input, ${\hat{\delta }}_{2}\;-{\hat{\delta }}_{1}$ = 0.045, 95% CI = [0.19, 0.71], which does not include 0. Unlike the vocabulary case presented earlier, no simplification of the model for syntax is justified. We have evidence that the coefficients for earlier input and later input are each nonzero for syntax, as neither CI includes 0 (see the 95% CI column in Table 3); it therefore does not make sense to estimate models in which either $\delta_{1}$ or $\delta_{2}$ is set to zero (an estimation that was warranted for vocabulary; see Rows 4 and 5 in Table 2). We also have evidence that the two effects differ; it therefore does not make sense to estimate a constant-effects model that constrains the effects to be equal (an estimation that was also warranted for vocabulary; see Row 3 in Table 2).

**Table 3.** Results of Weighted Outcome Model Estimating the Effect of Earlier (*Z*_1*S*_) and Later (*Z*_2*S*_ ) Parent Syntactic Input on Children’s Standardized CELF-RS Scores in Kindergarten

Model	Predictor	Coefficient estimate	β	95% CI	*SE*	*t* ratio	Nominal *p*
Differing effects	*Z*_1*V*_	${\hat{\delta }}_{1}$ = −0.24	−0.336	[−0.44, −0.03]	0.10	−2.36	.024
Differing effects	*Z*_2*V*_	${\hat{\delta }}_{2}$ = 0.22	0.446	[0.05, 0.38]	0.08	2.69	.014

Note: Results are based on five imputed data sets, with estimates and standard errors pooled according to Rubin’s rules (Rubin, 1987). CELF-RS = Clinical Evaluation of Language Fundamentals, Recalling Sentences subtest; CI = confidence interval.

Thus, for syntax, we estimated only one model: a differing-effects model, in which the coefficients for earlier and later input differ (Table 3). In this model, our estimate of the effect of earlier syntax input was negative, and our estimate of the effect of later syntax input was positive (see the β column in Table 3). This pattern suggests that (a) when syntax input later in development is taken into account, more complex early input is associated with significantly lower child syntax outcomes than less complex early input, and (b) when syntax input earlier in development is taken into account, more complex later input is associated with significantly higher child syntax outcomes than less complex later input. However, before we interpret this finding, it is important to look at the patterns of earlier and later input that we actually observed in our data and place the model results in this context. Our sensitivity analysis, reported in Section S8 in the Supplemental Material, suggests that reasonable levels of unobserved confounding would not lead to a bias of more than 18% of our estimates and would not qualitatively change our conclusions.

### Vocabulary and syntax compared

**Fig. 1.**
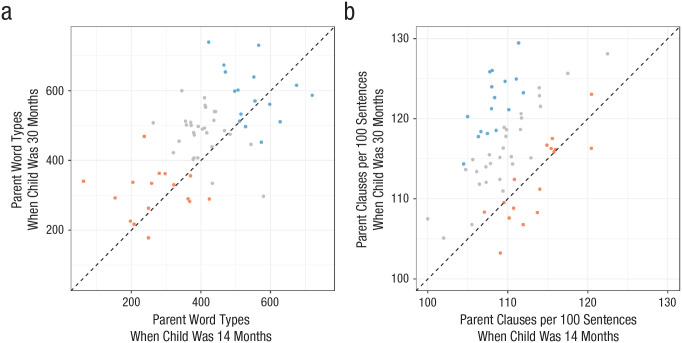
Scatterplots classifying each child’s predicted vocabulary outcome (a) and predicted syntactic outcome (b). Each point represents a child, plotted according to the parent’s input when the child was 14 months old (*x*-axis) and 30 months old (*y*-axis). Vocabulary input was measured as the number of unique word types the parent provided at each time point, and syntax input was measured as the number of clauses per 100 sentences the parent provided at each time point. Children’s vocabulary outcome was the Peabody Picture Vocabulary Test, and children’s syntax outcome was the Recalling Sentences subtest from the Clinical Evaluation of Language Fundamentals. Points in blue are children with the highest 16 predicted outcomes according to our best-fitting models (constant-effects model for vocabulary; differing-effects model for syntax). Points in red are children with the lowest 16 predicted outcomes; gray points are children in between. The dashed line shows where children would fall if their parents were perfectly stable in the input they provided (i.e., earlier and later input were identical).

To visually compare our best-fitting models for vocabulary and syntax, we first plotted each parent’s input for vocabulary (parent word types; Fig. 1a) and for syntax (parent clauses per 100 sentences; Fig. 1b) when their child was 14 months old and 30 months old. We then categorized each child’s predicted vocabulary outcome (PPVT) and predicted syntactic outcome (CELF-RS) using our best-fitting models (constant-effects model for vocabulary; differing-effects model for syntax). Children predicted by our models to have the highest outcomes for vocabulary or for syntax are highlighted in blue, children predicted to have middling outcomes are highlighted in gray, and children predicted to have the lowest outcomes are highlighted in red.

As our models suggested, we saw different patterns for vocabulary and syntax. First, it is important to note that parents’ word types tended to be stable across the two time points (the dots are arrayed along the dashed equality line in Fig. 1a), but their syntax tended to become more complex (the dots are above the dashed equality line in Fig. 1b). With respect to children’s outcomes, for vocabulary, children predicted to have the best outcomes (blue dots in Fig. 1a) were those whose parents’ input is in the top right of the graph, that is, parents who provided a diverse range of word types both earlier and later in the acquisition process. For syntax, children predicted to have the best outcomes (blue dots in Fig. 1b) were those whose parents are substantially above the equality line; that is, parents whose speech substantially increased in syntactic complexity when their child was 30 months old, compared with their baseline when the child was 14 months old. Children predicted to have the lowest outcomes for syntax (red dots) were those whose parents’ syntactic input remained stable, compared with their baseline, between when the children were 14 months and 30 months (points falling on the equality line). Interestingly, there were parents for whom the syntactic complexity of the input they give their children started high and remained high, but their children were predicted to have relatively low outcomes (the red dots in the upper right quadrant along the equality line). Note that few parents fell far below the equality line—in other words, it was rare for parents in our sample to provide substantially less complex input in absolute terms in either vocabulary or syntax when their child was 30 months old than when their child was 14 months old. Our findings should therefore not be interpreted as evidence that providing highly complex input too early actively harms children’s syntactic development. Rather, our data suggest that parents looking to support their children’s syntactic development should aim to increase the complexity of their own utterances over time.

Using our best-fitting model of the effects of vocabulary input, the constant-effects model reported in Row 3 of Table 2, we can calibrate the expected effect of different levels of cumulative input on child vocabulary outcomes. Consider, for example, a primary caregiver who provides vocabulary input of 336 word types when the child is 14 months old and 362 word types when the child is 30 months old (corresponding to the 25th percentile at both time points). For the child of this caregiver, our model predicts a PPVT score of 109.6. In contrast, consider a primary caregiver who provides vocabulary input of 474 word types when the child is 14 months old and 540 word types when the child is 30 months old (corresponding to the 75th percentile at both time points). For the child of this caregiver, our model predicts a PPVT score of 115.7. Referring to Table 1, we can see that the difference between the predicted PPVT scores of these two children is 0.46 of a standard deviation. To situate this finding in terms of previous results, consider that 0.46 is more than twice the average standardized effect of state prekindergarten intervention programs on PPVT (Wong et al., 2008).

For syntax, we found that caregivers whose speech increased in absolute complexity between when their child was 14 and 30 months old generated favorable syntax outcomes in their children. We can calibrate the impact of sequences of input by applying our best-fitting model for syntax (a differing-effects model; Table 3). Consider, for example, two caregivers, each of whom produces 108 clauses per 100 utterances when the child is 14 months old (corresponding to the 25th percentile). Now suppose that one caregiver’s input is roughly the same when the child is 30 months old—112 clauses per 100 utterances (corresponding again to the 25th percentile). Our model predicts a CELF-RS score of 10.5 for the child of this caregiver. In contrast, the second caregiver’s syntactic input increases when the child is 30 months old to 121 clauses per 100 utterances (corresponding to the 75th percentile). Our model predicts a CELF-RS score of 12.4 for the child of this caregiver. Referring to Table 1 to find the standard deviation of CELF-RS score, we computed a difference score between the expected outcomes for these two children and found that the difference was 0.63 of a standard deviation, which is a large effect.

## Discussion

Parents provide varying language experiences to their children. This variability has catalyzed decades of research examining how the input that parents provide shapes children’s language development. Previous work has found a strong relation between early parent input and child vocabulary at school entrance (Hoff, 2003; Huttenlocher et al., 1991; Rowe et al., 2009; Rowe & Goldin-Meadow, 2009; Weizman & Snow, 2001) but contradictory results on child syntax (Gleitman et al., 1984; Huttenlocher et al., 2002; Scarborough & Wyckoff, 1986). These findings are open to multiple interpretations. First, earlier parent input could be responsible for the relation between parent input and later child outcomes, as has been assumed. Alternatively, later input could also be responsible for this relation, because parents who use rich input when speaking to their child early in development are likely to continue to do so. The third possibility is that the sequence of input over time, not input at a particular time point, could be responsible for later child outcomes. Our goal was to distinguish among these hypotheses and thus determine whether the impact of parent language input varies as a function of developmental time. If it does, we also asked whether these timing effects differ for vocabulary and syntax acquisition.

We first replicated the effects found in the literature. Early parent input in our data predicted later child outcomes with respect to vocabulary but not with respect to syntax. We then assessed the impact of sequences of input when children were 14 and 30 months old and found different answers for vocabulary and syntax. For vocabulary, the most parsimonious explanation of our data was that providing a diverse range of parent word types earlier and later in the acquisition process predicts the best child outcomes. In contrast, for syntax, the most parsimonious explanation of our data was that the effects of earlier and later parent input vary and that sequencing of the input matters; the optimal sequence was for parents’ absolute level of syntactic complexity of input to increase over time rather than simply remain stable.

Why might timing effects differ for vocabulary and syntax? Learning the lexical items of a language requires a great deal of data from that language—according to Cristia (2020), it is a data-hungry process. Consistent with this view, our results showed that high levels of vocabulary input were beneficial both earlier and later in development. Learning other aspects of language may be supported by strong prior knowledge and thus may not require as much input (Cristia, 2020); for example, deaf children who are not exposed to sign language nevertheless develop the ability to produce sentences containing multiple verbs, showing that this syntactic skill can emerge without any linguistic input whatsoever (Goldin-Meadow, 2020; Goldin-Meadow & Mylander, 1998). But linguistic input is clearly essential when it comes time for children to learn how to produce multiple-verb sentences in the language of their community. We did find that input matters for syntax, but child outcomes seemed to depend less on the absolute complexity of syntax input at each time point than on the extent to which input complexity increased over time. One possibility is that this increase in complexity in the input itself functions as a cue, prompting children to attend to and acquire these and other syntactic structures at this later point in language development. Another possibility is that although diverse vocabulary input is useful to children from the beginning of language development, complex syntax input is useful only once children’s language reaches a sufficient level of development (Vygotsky, 1978).

Our results underscore the need for novel approaches that account for the dynamic relations between parents and children. Recent examinations of the role of parent language input highlight the predictive power of both quantitative and qualitative differences in parent input (e.g., Cartmill et al., 2013). Our findings suggest that characterizing static features of input, quantity or quality, at a single time point provides a limited view. We should instead characterize features of input dynamically at multiple levels of analysis and multiple time points (e.g., Irvin et al., 2016).

Our study is unique in bringing methods from epidemiology into language acquisition, enabling us to adjust for time-invariant and time-varying confounding and thus estimate the true impact of parent input on child outcomes. These methods allowed us to account for important confounders known to influence this relation.^1^ However, we cannot rule out the potential influence of confounders we did not directly observe; for example, genetic factors not captured by parent verbal IQ, prenatal environment, or environmental stressors. In addition, our results were restricted to a North American sample and thus need to be extended to cultures in which children receive less child-directed speech from their parents (Casillas et al., 2020). The differences we observed between input effects on vocabulary and syntax in English may not replicate in children acquiring other languages, particularly those with more complex morphosyntax. Finally, although our work constitutes an advance in showing that later input matters even when earlier input is accounted for, we cannot rule out effects of input measured still later (after 30 months). Future work should focus on a wider range of input time points to obtain a richer picture of the optimal sequencing of language input.

The novel approach we employed here is well suited to exploring the dynamic relations between parent language input and child language outcomes. Our hope is that this approach will be extended to other areas—for example, to the effects of stress, instruction, or therapy—in which it is essential to understand the impact of continuously measured, time-varying exposures.

## Supplemental Material

sj-docx-1-pss-10.1177_0956797620970559 – Supplemental material for Effects of Time-Varying Parent Input on Children’s Language Outcomes Differ for Vocabulary and Syntax Click here for additional data file. Supplemental material, sj-docx-1-pss-10.1177_0956797620970559 for Effects of Time-Varying Parent Input on Children’s Language Outcomes Differ for Vocabulary and Syntax by Catriona Silvey, Özlem Ece Demir-Lira, Susan Goldin-Meadow and Stephen W. Raudenbush in Psychological Science

## Transparency

*Action Editor:* Rebecca Treiman

*Editor:* D. Stephen Lindsay

*Author Contributions*

S. W. Raudenbush and S. Goldin-Meadow conceived the study. S. Goldin-Meadow was in charge of collecting the longitudinal data. C. Silvey and Ö. E. Demir-Lira analyzed the data in consultation with S. W. Raudenbush. S. W. Raudenbush developed the method for the sensitivity analysis and derived the proofs. C. Silvey developed and coded the simulations for determining number of quantiles and for testing the robustness of the method in small samples. C. Silvey and Ö. E. Demir-Lira drafted the manuscript, and all authors provided critical revisions. All authors approved the final version of the manuscript for submission.

**Open Practices:** Code for the data analysis and simulations is provided on GitHub at https://github.com/silveycat/vocab-syntax. Data cannot be publicly shared because the relevant permissions were not obtained from participants when the data were originally collected. Materials for this study have not been made publicly available, and the design and analysis plans were not preregistered.

## Notes

1. As discussed in the Method section, because we used weighting to control for child speech as a time-varying confounder, our model controlled for the extent to which parents tailor their later input to the child’s response to earlier input. If we could randomly assign children to sequences of input, we would not need this control.
