# Supplementary material for: Effects of Time-Varying Parent Input on Children’s Language Outcomes Differ for Vocabulary and Syntax
Source: Psychol Sci. 2021 Mar 15;32(4):536–48. doi: 10.1177/0956797620970559 (PMC8726591; doi:10.1177/0956797620970559)
Supplement: sj-docx-1-pss-10.1177_0956797620970559 – Supplemental material for Effects of Time-Varying Parent Input on Children’s Language Outcomes Differ for Vocabulary and Syntax [file sj-docx-1-pss-10.1177_0956797620970559.docx]

**Supplemental Material**

**Section S1.** Model of growth in PPVT scores used to estimate vocabulary outcome

To obtain a reliable estimate of children’s vocabulary status in kindergarten, we built a two-level statistical model for child-specific growth in standardized PPVT scores using hierarchical linear modeling (HLM; Raudenbush & Bryk, 2002). Outcomes were standardized PPVT scores measured at 5 time points: when children were 42 months old, 54 months old, in preschool, in kindergarten, and in 2^nd^ grade.

The Level 1 model accounted for the variation in repeated measures of vocabulary within each child. The Level 2 model accounted for the variation between children. We used child age in months at each visit as our measure of time and centered age at 74 months, which is the average age at which CELF measures were administered.

At Level 1 (within children), we represented children’s vocabulary trajectory using a linear model. So for each child, we had:

$Y_{ti}=\pi_{0i}+\pi_{1i}*(a_{ti}-74)+e_{ti},e_{ti}\sim N(0,s_{t}^{2})$ (S1)

In this equation, *a_ti_* is the age in months of child *i* at time *t*, $\pi_{0i}$ is child *i*'s status (average standardized PPVT score) at age 74 months, and $\pi_{1i}$ is child *i*'s growth velocity at age 74 months. The residual $e_{ti}$ represents that portion of child *i*'s standardized PPVT score at age *t* that is not predicted by his or her age. We assume a heterogeneous level-q variance, meaning that the within-person variation varies over time with child age. We determined that the linear model best represented the empirical data because it had a better goodness-of-fit statistic (-2 log likelihood) than the quadratic model and because the plot of the linear model best mirrored the plot of the empirical data.

We had a separate Level 2 equation for each Level 1 coefficient, $\pi_{pi}$, where p = 0, 1:

$\pi_{pi}=\beta_{p0}+r_{pi}$ (S2)

where *π_pi_* is the p^th^ growth parameter from the Level 1 model and *r_pi_* is a random effect. From this model, we extracted the OLS estimate of the random intercept $\pi_{0i}$ for all children who had sufficient data. For 2 children who only had one data point, we extracted the empirical Bayes estimate of $\pi_{0i}$. These estimates were then used as our outcome variable for vocabulary. The reliability estimate for $\pi_{0i}$was .910, suggesting that this process gives us a highly reliable estimate of children’s standardized PPVT scores at 74 months.

The linear model showed that, on average, at age 74 months children had an estimated standardized PPVT score of approximately 109 (SE 1.89, *p* < .001), with an increase in standardized scores at this age at a rate of 0.059 points per month (SE 0.026, *p* = .022). There was significant variation in children’s intercepts, SD = 14.0, *p* < .001, but not in children’s slopes, SD = 0.062, *p* = .442. The deviance of the model was 1964.28.

**Section S2.** Multiple imputation procedure

To impute missing data, we used the mice (Multivariate Imputation by Chained Equations) package in R (van Buuren & Groothuis-Oudshoorn, 2011). The method uses predictive mean matching (Little, 1988; Rubin, 1986). Predictive mean matching calculates the predicted value of each variable in turn on the basis of all other variables. Besides the variables listed in Table 1, we used the following auxiliary variables to increase the robustness of our imputation: child age in months at onset of producing multi-clause utterances; child word types at 22 months; child mean length of utterance at 22 months; parent word types at 26 months; parent mean length of utterance at 26 months; child PPVT scores at 30, 42, and 54 months; child word types at 46 months; child average clauses per sentence across 46 and 50 month sessions; and child sentence repetition score from the GAPS (Grammar and Phonology Screening: Gardner, Froud, McClelland, & van der Lely, 2006) administered at 46 months. For each missing data point, the 5 complete cases whose predicted values are closest to the predicted value of the missing data point are selected as candidate ‘donors’. One of these donors is then selected at random, and the observed value for the donor case is used to replace the missing data point (van Buuren, 2018). Five complete data sets were imputed using this method. One complete data set was used to assess covariates for inclusion (see Text S6) and to run our sensitivity analysis (see Text S8). Weights were generated individually for each imputed data set using the same weight-generating model. We estimated weighted outcome models on each of the five imputed data sets and the results were pooled according to Rubin’s rules (Rubin, 1987).

We can also run an alternative analysis that uses only complete data. However, if we simply drop incomplete cases, we run the risk of introducing bias, since missing data are likely not missing at random: the probability of a case being complete may be related to our covariates. Therefore, we must create additional weights that adjust for the probability of missingness.

To do this, we first calculate our combined weights $W_{1,2}$ as detailed in Text S3. We then create an indicator variable which equals 0 where any variable is missing for that case, and 1 otherwise. We run a logistic regression predicting this indicator from all variables in our dataset for which we have no missing values. From this model, we predict the probability for each case of having complete data. We then calculate a missingness weight $W_{m}$ , where the numerator is the overall proportion of cases that are complete and the denominator is the case-specific probability of completeness given covariates. The resulting weight will be > 1 for children who are relatively unlikely, given their covariates, to have complete data, and < 1 for children who are relatively likely, given their covariates, to have complete data. We then multiply our combined weights $W_{1,2}$ by missingness weights $W_{m}$ to produce final weights $W_{f}$ and run the same outcome models we report in Tables 2 and 3 on complete cases only using weights $W_{f}$ .

The results are reported in Tables S1 and S2. The model estimates are very similar to those we obtain when using imputation. Two differences emerge: (1) for vocabulary, AIC now slightly favors the model where later input is sufficient over the constant effects model; (2) for syntax, the effect of later input is no longer significant at *p* < .05. Since using only complete cases reduces the power of our analyses, these differences are not entirely unexpected, and we choose to report the main results from our more highly powered imputation-based analysis. However, it is reassuring that the results are broadly similar under both approaches. Code for the complete cases analysis is provided in the file analysis_code_missing.R at <https://github.com/silveycat/vocab-syntax>.

**Table S1.** Outcome models for vocabulary using only complete cases and weights $W_{f}$, generated as described in Text S2.

| Model | Predictor | Coefficient  Estimate | 95% CI | Standard error | *t*-ratio | Nominal *p* value | AICc |
| --- | --- | --- | --- | --- | --- | --- | --- |
| Differing effects earlier and later | $Z_{1V}$ $Z_{2V}$ | $\hat{\delta}_{1}$=0.008  $\hat{\delta}_{2}$=0.036 | [-0.018, 0.034]  [0.003, 0.070] | 0.013  0.017 | 0.60  2.17 | .552  .035 | 375.5 |
| Constant effects | $Z_{1V}+Z_{2V}$ | $\hat{\delta}$=0.022 | [0.007, 0.038] | 0.008 | 2.91 | .006 | 374.3 |
| Earlier input is sufficient | $Z_{1V}$ | $\hat{\delta}_{1}$=0.025 | [-0.002, 0.053] | 0.014 | 1.86 | .069 | 378.8 |
| Later input is sufficient | $Z_{2V}$ | $\hat{\delta}_{2}$=0.040 | [0.009, 0.070] | 0.015 | 2.64 | .011 | 373.4 |

**Table S2.** Outcome model for syntax using only complete cases with weights $W_{f}$, generated as described in Text S2.

| Model | Predictor | Coefficient  Estimate | 95% CI | Standard error | *t*-ratio | Nominal *p* value |
| --- | --- | --- | --- | --- | --- | --- |
| Differing effects earlier and later | $Z_{1V}$ $Z_{2V}$ | $\hat{\delta}_{1}$=-0.29  $\hat{\delta}_{2}$=0.24 | [-0.57, -0.02]  [-0.01, 0.49] | 0.14  0.13 | -2.14  1.93 | .039  .061 |

**Section S3.** Full details of analytic method

The quantile binning method as applied to our data involves the following steps. (When referring to quantiles, uppercase *M* is the number of quantiles and lowercase *m* is the specific quantile a child was assigned to.)

1) Bin sample into *M* quantiles on the basis of continuous earlier input $Z_{1}$ . Given our sample size of N=64, we chose *M*=8 quantiles, each of size 8, a choice justified by simulations reported in Text S4 and Figures S1 and S2 below.

2) Predict quantile $\Pr(R=m|X_{0}),m=1,...M$where *R* is the ordinal response and $X_{0}$ is a vector of baseline covariates. This procedure allows us to estimate how likely each child was to receive the actual level of earlier input the child received, given that child’s baseline covariates.

3) Calculate weights $W_{1}$. The numerator of each weight is constant: it equals the marginal probability of being assigned to quantile *m*, that is, $\Pr(R=m)=1/M=1/8$. The denominator varies for each case: it equals the predicted probability of being assigned to quantile *m* on the basis of covariates, that is,$\Pr(R=m|X_{0})$, derived from the ordinal regression in step 2. This means that children who were relatively likely to receive the actual level of input they received will be down-weighted (since the denominator will be larger), whereas children who were relatively unlikely to receive the actual level of input they received will be up-weighted (since the denominator will be smaller). This procedure allows us to use weighting to account for confounding.

4) Bin sample into *Q* quantiles on the basis of continuous later input $Z_{2}$ .

5) Predict quantile $\Pr(S=q|X_{0},X_{1},Z_{1}),q=1,...Q$, where *S* is the ordinal response, $X_{0}$ is a vector of baseline covariates, $X_{1}$ is the time-varying covariate (child language at 26 months), and $Z_{1}$ is earlier input. This procedure allows us to estimate how likely each child was to receive the actual level of later input the child received, given that child’s baseline and time-varying covariates and level of earlier input.

6) Calculate weights $W_{2}$. The numerator of each weight equals the predicted probability of being assigned to quantile *n* on the basis of earlier input, that is, $\Pr(S=q|Z_{1})$, derived from an ordinal regression where the outcome is the quantile *n* and the predictor is earlier input $Z_{1}$. The denominator equals the predicted probability of being assigned to quantile *n* on the basis of baseline and time-varying covariates and earlier input, that is, $\Pr(S=q|X_{0},X_{1},Z_{1})$, derived from the ordinal regression in step 5.

7) Once weights $W_{1}$ and $W_{2}$ have been calculated, multiply these weights to create combined weights $W_{1,2}$ and estimate the association between $Z_{1}$, $Z_{2}$ and $Y$ using weighted least squares.

In the absence of random assignment of children to sequences of parent input, we assume that elimination of confounding attributable to our measured covariates is sufficient to remove any correlation between the error and the inputs.

Note that while our principal models reported in Tables 2 and 3 use combined weights , our preliminary models reported in the text that estimate equation 2 (replicating previous analyses by estimating the apparent effect of earlier input without controlling for later input) use weights $W_{1}$ .

**Section S4.** Choosing number of quantiles

In choosing the number of quantiles for our analysis, we have two aims: (1) to minimize the bias in the estimates, i.e., ensure our estimate for each parameter is as close as possible to the true population value; (2) to minimize the variance in the generated weights, i.e., ensure our analysis is stable. As the number of quantiles increases, we expect the bias to decrease, but the variance of the weights to increase. Our aim was to find a ‘happy medium’ number of quantiles that would minimize both the bias and the variance in the weights. We ran a simple simulation using the quantile binning method to estimate the effect of a continuous treatment *Z* on an outcome *Y*, where a confounder *X* was associated with both *Z* and *Y*. The sample size was the same as in our own data, *N* = 64. We ran the simulation 1000 times for every number of quantiles from 3 to 64. Figures S1 and S2 below show the bias and variance of the weights, averaged across the 1000 runs. We can see that our choice of 8 quantiles is a good compromise, associated with a relatively low bias but also low variance in weights. Code for the simulation is provided in the file strata_sim.R at <https://github.com/silveycat/vocab-syntax>.


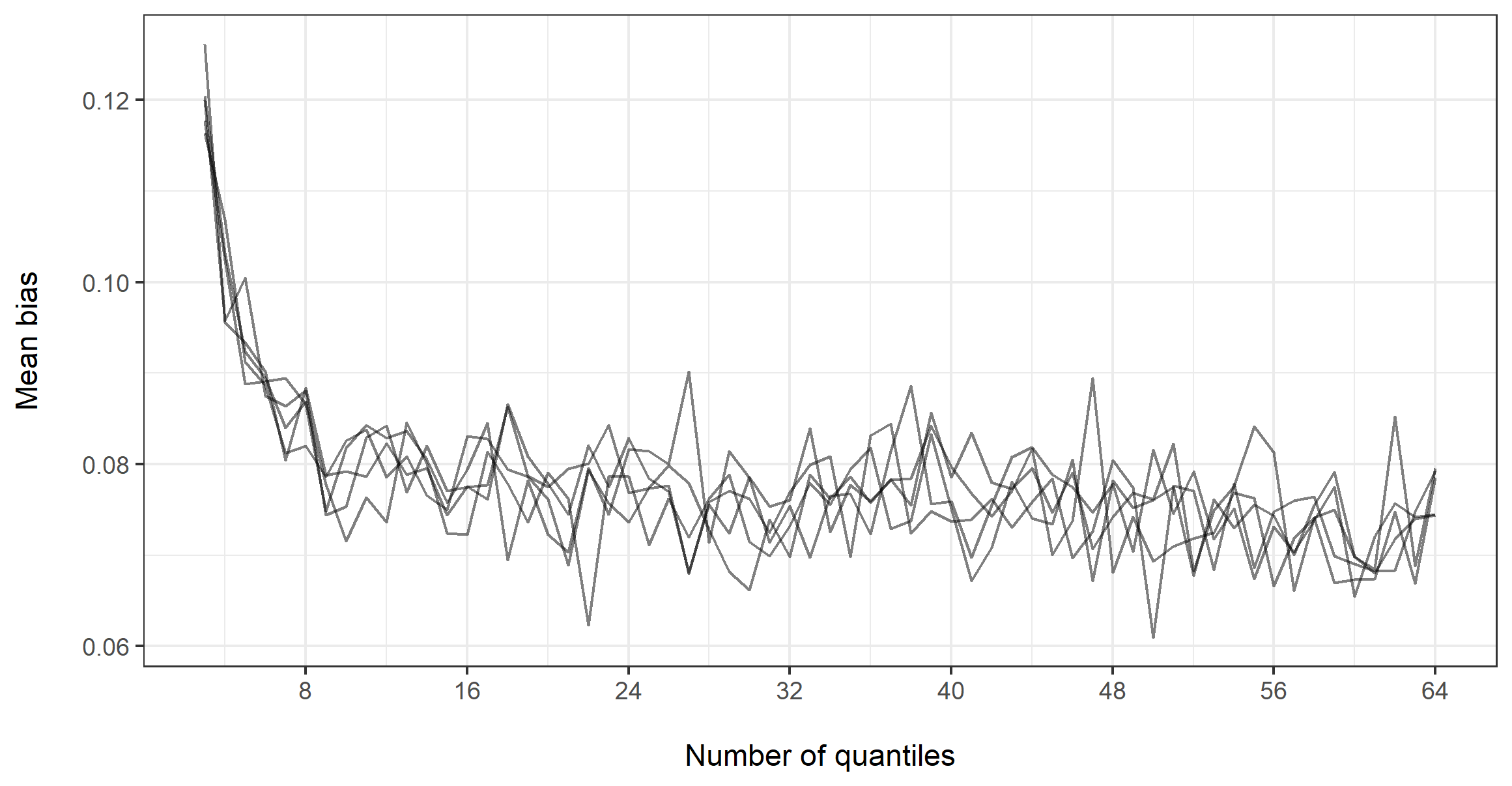


**Figure S1.** Bias of estimates from weighted outcome models with differing number of quantiles. Separate lines show five separate runs of the simulation.

**
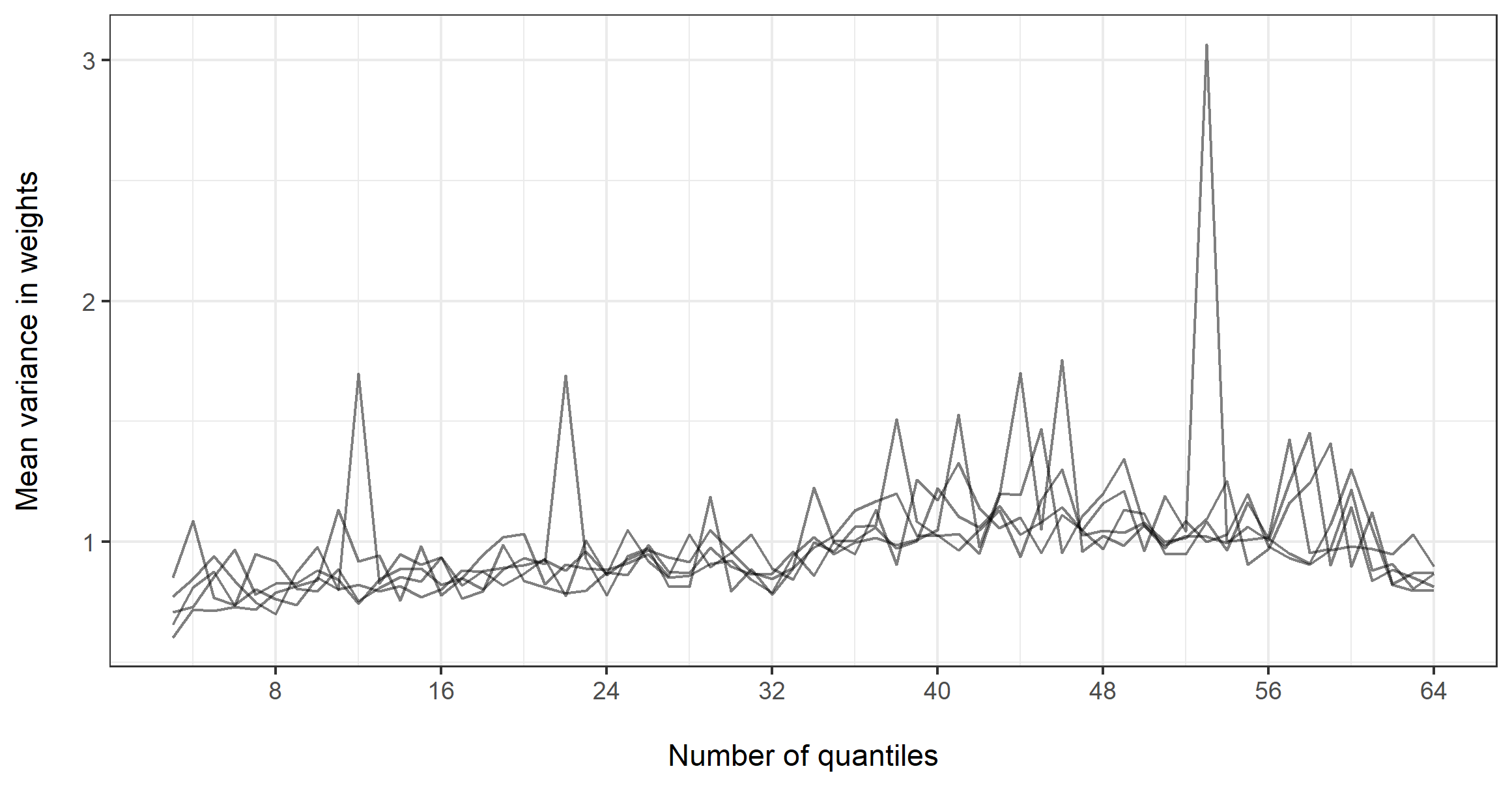
**

**Figure S2.** Average variance of weights with differing number of quantiles. Separate lines show five separate runs of the simulation.

**Section S5.** Assessing robustness of the quantile binning method in a small sample

Although Naimi et al. (2014) demonstrate robust results using the quantile binning method, their study simulates very large datasets. In our study, we have a relatively small sample of 64 parents and children. A natural question is whether we can expect the quantile binning method to produce similarly robust results in a small sample.

We can address this question by simulating data where a true effect exists, and then testing how well we recover this effect when we apply the quantile binning method to samples of different sizes. From these simulations, we can assess (1) the bias, or the average error in estimating an effect, and (2) the correspondence between the estimated standard error reported by our models and the empirical standard error (i.e., the standard deviation of the actual errors in estimation when the analysis is repeated across many samples). For (1), if the bias is worse in small samples than in large samples, this result might suggest the method is inappropriate for use with small samples. For (2), although our estimates will naturally have larger standard errors when we analyze a smaller sample, we want to ensure that the standard error estimated by our models is not likely to be an underestimate of the true amount of uncertainty.

To answer these questions, we adapt the simulation we used for determining the optimal number of strata, described in Text S4, and vary the sample size. We simulate and analyze 1000 datasets with a sample size of 64 (matching our data), and 1000 datasets with a larger sample size of 500. We set the true effect size to be 0.3, similar to the effect we find in our cumulative vocabulary analysis.

We calculate the bias by averaging across the 1000 analyses the difference between the true population effect and the effect estimated by our models. For the small samples, we find a bias of -0.04; for the large samples, we find a bias of -0.07. We therefore do not find a systematically larger bias in small samples using this method. This result suggests that for data like ours, the quantile binning method may actually be slightly conservative; if anything, we are likely to be overestimating, rather than underestimating, any effects.

We also compare the average standard error estimated across each of the 1000 analyses with the empirical standard error (i.e., the standard deviation of the differences between the true and estimated effect across all analyses). For the small samples, we find an empirical standard error of 0.124 and an average estimated standard error of 0.127; for the large samples, we find an empirical standard error of 0.049 and an average estimated standard error of 0.052. For both small and large samples, the standard error estimated by the models is an accurate estimate of the true amount of uncertainty.

Code for the simulation is provided in the file sample_sim.R at <https://github.com/silveycat/vocab-syntax>.

**Section S6.** Assessing covariates for inclusion and checking balance

Our sample is relatively small (64) and our number of covariates relatively large (7 baseline covariates $X_{0}$ and 1 time-varying covariate $X_{1}$). We therefore chose not to include all covariates in our weight-generating models. Instead, we used a step-wise procedure to assess covariates for inclusion. We checked balance on all observed covariates after weighting, including those not in our weight-generating model, to ensure that we eliminated all observed confounding. See Tables S3-S6 for the full balance checks.

We assessed covariates for inclusion and checked balance using the following steps:

1) Run unweighted linear regressions predicting each covariate $X_{0}$ from $Z_{1}$. From each regression, record the standardized coefficient of $Z_{1}$ and the associated *t*-value. Report these values as the associations between each covariate and $Z_{1}$ before weighting (see Table S3 for vocabulary, and Table S5 for syntax).

2) Select the covariate most strongly associated with $Z_{1}$, i.e., with the largest *t*-ratio.

3) Generate weights $W_{1}$ on the basis of this covariate, as detailed in steps 2 and 3 of Text S3.

4) Run weighted linear regressions using weights $W_{1}$, predicting each covariate $X_{0}$ (including those not in the weight-generating model) from $Z_{1}$.

5) If any covariates remain associated with $Z_{1}$ at *t* > 1.67 or *t* < -1.67, add the covariate with the largest *t*-ratio to the weight-generating model.

6) Repeat steps 3-5 until -1.67 < *t* < 1.67 for all covariates.

7) From each final weighted regression, record the standardized coefficient of $Z_{1}$ and the associated *t*-value. Report these values as the associations between each covariate and $Z_{1}$ after weighting (see Table S3 for vocabulary, and Table S5 for syntax).

8) Continue for $Z_{2}$. Run unweighted linear regressions predicting each covariate $X_{0},X_{1}$ from $Z_{1}$ and $Z_{2}$ as separate predictors in the same model. From each regression, record the standardized coefficient of $Z_{2}$ and the associated *t*-value. Report these values as the associations between each covariate and $Z_{2}$ before weighting (see Table S4 for vocabulary, and Table S6 for syntax). Note that covariates’ associations with $Z_{1}$ can be ignored for the purposes of balance-checking.

9) Set weights $W_{2}$ initially equal to 1, such that combined weights $W_{1,2}=W_{1}$ .

10) Run weighted linear regressions using weights $W_{1,2}$ predicting each covariate $X_{0},X_{1}$ from $Z_{1}$ and $Z_{2}$ as separate predictors in the same model.

11) Select the covariate most strongly associated with $Z_{2}$, i.e., with the largest *t*-ratio.

12) Generate weights $W_{2}$on the basis of this covariate, as detailed in steps 5 and 6 of Text S3.

13) Multiply weights $W_{1}$ and $W_{2}$ to obtain combined weights $W_{1,2}$ .

14) Run weighted linear regressions using combined weights $W_{1,2}$ predicting each covariate $X_{0},X_{1}$ from $Z_{1}$ and $Z_{2}$ as separate predictors in the same model. If any covariates remain associated with $Z_{2}$ at *t* > 1.67 or *t* < -1.67, add the covariate with the largest *t*-ratio to the weight-generating model.

15) Repeat steps 12-14 until -1.67 < *t* < 1.67 for all covariates’ associations with $Z_{2}$.

16) From each final weighted regression, record the standardized coefficient of $Z_{2}$ and the associated *t*-value. Report these values as the associations between each covariate and $Z_{2}$ after weighting (see Table S4 for vocabulary, and Table S6 for syntax).

The covariates included in the final weight-generating models are:

$Z_{1V}$: parent verbal IQ, household income, and child gender

$Z_{2V}$: parent word types at 14 months ($Z_{1V}$) and child language at 26 months ($X_{1}$)

$Z_{1S}$: parent verbal IQ, child birth order, and child word types at 14 months

$Z_{2S}$: parent clauses per sentence at 14 months ($Z_{1S}$), child language at 26 months ($X_{1}$), parent verbal IQ, parent education, and child gender

By including these covariates in our weight-generating models, we are able to achieve balance on all observed covariates. Tables S3-S6 below report standardized coefficients and *t*-values before and after weighting for $Z_{1}$ and $Z_{2}$ for both vocabulary and syntax.

**Table S3.** Bivariate relations between $Z_{1V}$ and baseline covariates $X_{0}$, before and after weighting.^a^.

| **Variable** | **Before weighting** | | **After weighting** | | |
| --- | --- | --- | --- | --- | --- |
|  | **Standardized coefficient** | ***t*-ratio** | | **Standardized coefficient** | ***t*-ratio** | |
| Child birth order | -0.02 | -0.13 | | 0.05 | 0.35 | |
| Child gender | -0.13 | -1.12 | | -0.18 | -1.29 | |
| Child word types at 14 months | -0.05 | -0.47 | | -0.02 | -0.14 | |
| Child gesture types at 14 months | 0.18 | 1.76 | | 0.13 | 1.04 | |
| Parent verbal IQ | 0.37 | 3.27 | | 0.11 | 0.78 | |
| Parent education | 0.35 | 2.66 | | 0.05 | 0.35 | |
| Household income | 0.39 | 3.41 | | 0.07 | 0.41 | |

^a^. Standardized coefficients and *t*-ratios are reported from regressions predicting each covariate $X_{0}$ from $Z_{1V}$, without weighting (columns 2 and 3) and with weighting (columns 4 and 5).

**Table S4.** Bivariate relations between $Z_{2V}$, baseline covariates $X_{0}$, and time-varying covariate $X_{1}$, before and after weighting.^a^.

| **Variable** | **Before weighting** | | **After weighting** | |
| --- | --- | --- | --- | --- |
|  | **Standardized coefficient** | ***t*-ratio** | **Standardized coefficient** | ***t*-ratio** |
| Child language 26 months $X_{1}$ | 0.31 | 2.03 | 0.08 | 0.52 |
| Child birth order | 0.06 | 0.37 | -0.09 | -0.50 |
| Child gender | -0.08 | -0.47 | -0.05 | -0.30 |
| Child word types at 14 months | 0.12 | 0.74 | 0.07 | 0.39 |
| Child gesture types at 14 months | 0.13 | 0.77 | 0.05 | 0.22 |
| Parent verbal IQ | 0.08 | 0.48 | 0.14 | 0.70 |
| Parent education | 0.15 | 0.79 | 0.24 | 1.14 |
| Household income | 0.24 | 1.82 | 0.18 | 1.04 |

^a^. Standardized coefficients and *t*-ratios are reported from regressions predicting each variable from $Z_{1V}$and$Z_{2V}$, without weighting (columns 2 and 3) and with weighting (columns 4 and 5).

**Table S5.** Bivariate relations between $Z_{1S}$ and baseline covariates $X_{0}$, before and after weighting.^a^.

| **Variable** | **Before weighting** | | **After weighting** | |
| --- | --- | --- | --- | --- |
|  | **Standardized coefficient** | ***t*-ratio** | **Standardized coefficient** | ***t*-ratio** |
| Child birth order | -0.23 | -2.14 | -0.13 | -1.00 |
| Child gender | 0.13 | 1.03 | 0.16 | 1.15 |
| Child word types at 14 months | -0.24 | -1.99 | -0.05 | -0.40 |
| Child gesture types at 14 months | -0.10 | -0.83 | 0.00 | 0.01 |
| Parent verbal IQ | 0.29 | 2.64 | 0.14 | 1.22 |
| Parent education | 0.10 | 0.80 | 0.04 | 0.32 |
| Household income | 0.00 | 0.03 | -0.05 | -0.37 |

^a^. Standardized coefficients and *t*-ratios are reported from regressions predicting each covariate $X_{0}$ from $Z_{1S}$, without weighting (columns 2 and 3) and with weighting (columns 4 and 5).

**Table S6.** Bivariate relations between $Z_{2S}$ and baseline covariates $X_{0}$ and time-varying covariate $X_{1}$, before and after weighting.^a^.

| **Variable** | **Before weighting** | | **After weighting** | |
| --- | --- | --- | --- | --- |
|  | **Standardized coefficient** | ***t*-ratio** | **Standardized coefficient** | ***t*-ratio** |
| Child language 26 months $X_{1}$ | 0.44 | 4.09 | 0.11 | 0.69 |
| Child birth order | 0.17 | 1.33 | -0.03 | -0.19 |
| Child gender | -0.21 | -1.74 | -0.09 | -0.57 |
| Child word types at 14 months | 0.05 | 0.34 | -0.01 | -0.08 |
| Child gesture types at 14 months | 0.18 | 1.41 | 0.07 | 0.46 |
| Parent verbal IQ | 0.41 | 3.30 | 0.22 | 1.27 |
| Parent education | 0.29 | 2.06 | 0.12 | 0.83 |
| Household income | 0.15 | 1.19 | 0.11 | 0.71 |

^a^. Standardized coefficients and *t*-ratios are reported from regressions predicting each variable from $Z_{2S}$, without weighting (columns 2 and 3) and with weighting (columns 4 and 5).

**Section S7.** Common support

Common support is present when subsets of children who have the same or similar probability of a given level of input vary substantially on the actual input they received (Hong, 2012). Lack of common support arises when children who have the same or similar probability of a given level of input vary little. Lack of common support implies that some subsets of children contain no information about the impact of input (e.g., those with high input have no comparison group).

Figures S3-S6 show the distribution of observed input (divided into 8 quantiles) for each of 4 categories of children: those predicted, on the basis of their covariates, to have low input (quantile 1-2), those predicted to have low-mid input (quantile 3-4), those predicted to have mid-high input (quantile 5-6), and those predicted to have high input (quantile 7-8). We consider common support achieved where there is a good range of observed input for children in each category of predicted input.


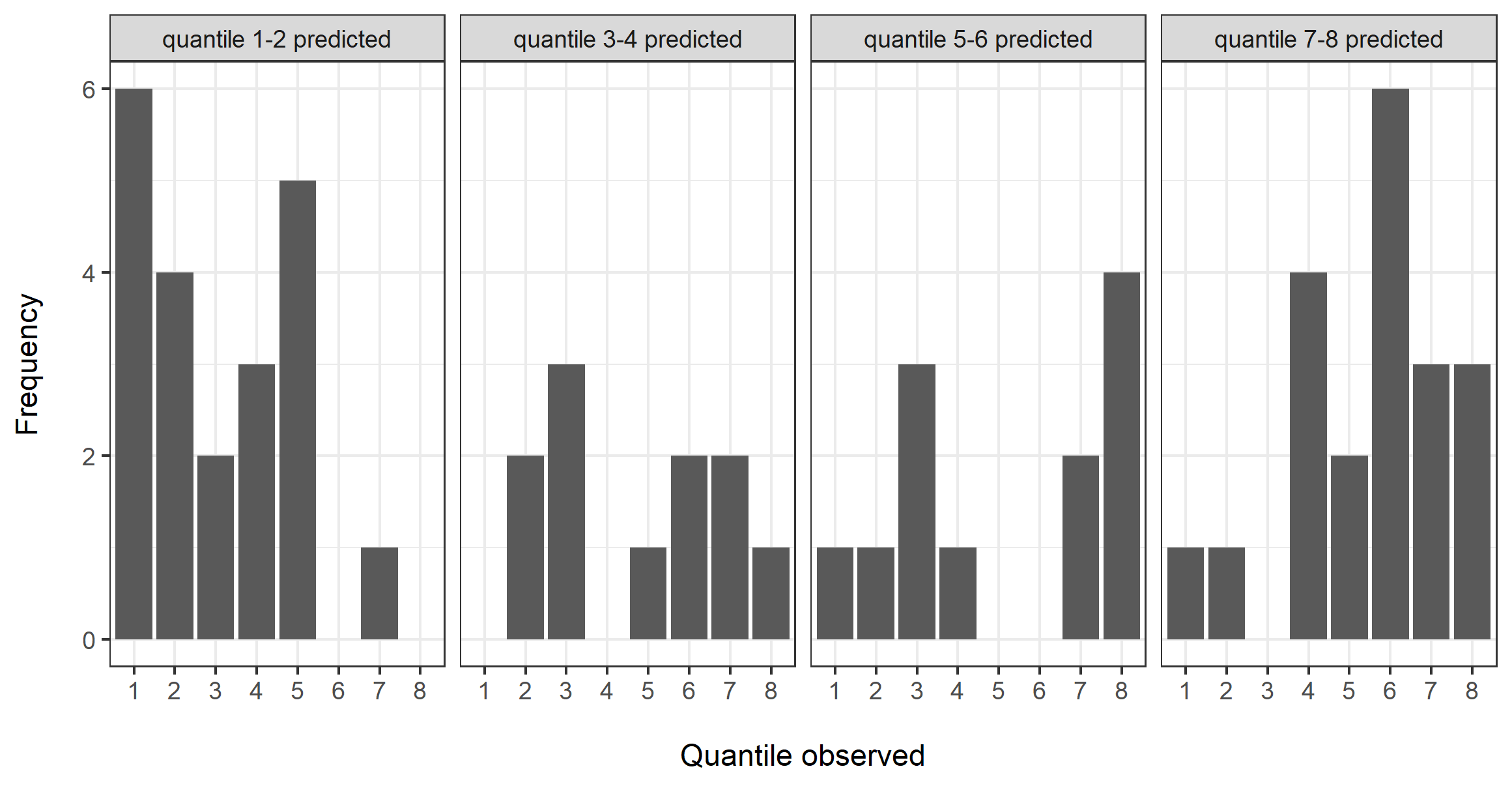


**Figure S3.** Common support for estimating the effect of earlier vocabulary input $Z_{1V}$(parent word types at 14 months).


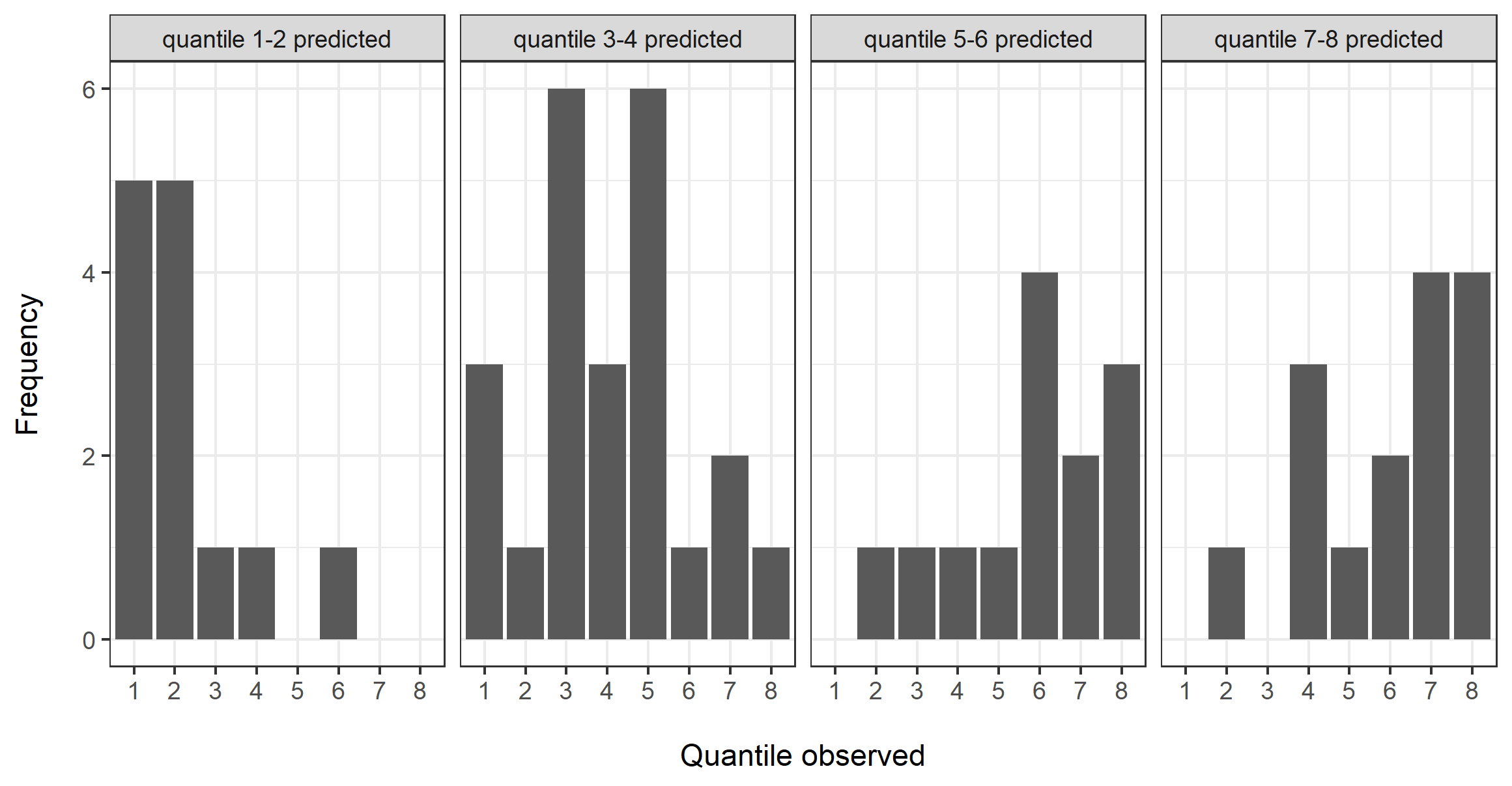


**Figure S4.** Common support for estimating the effect of later vocabulary input $Z_{2V}$(parent word types at 30 months).


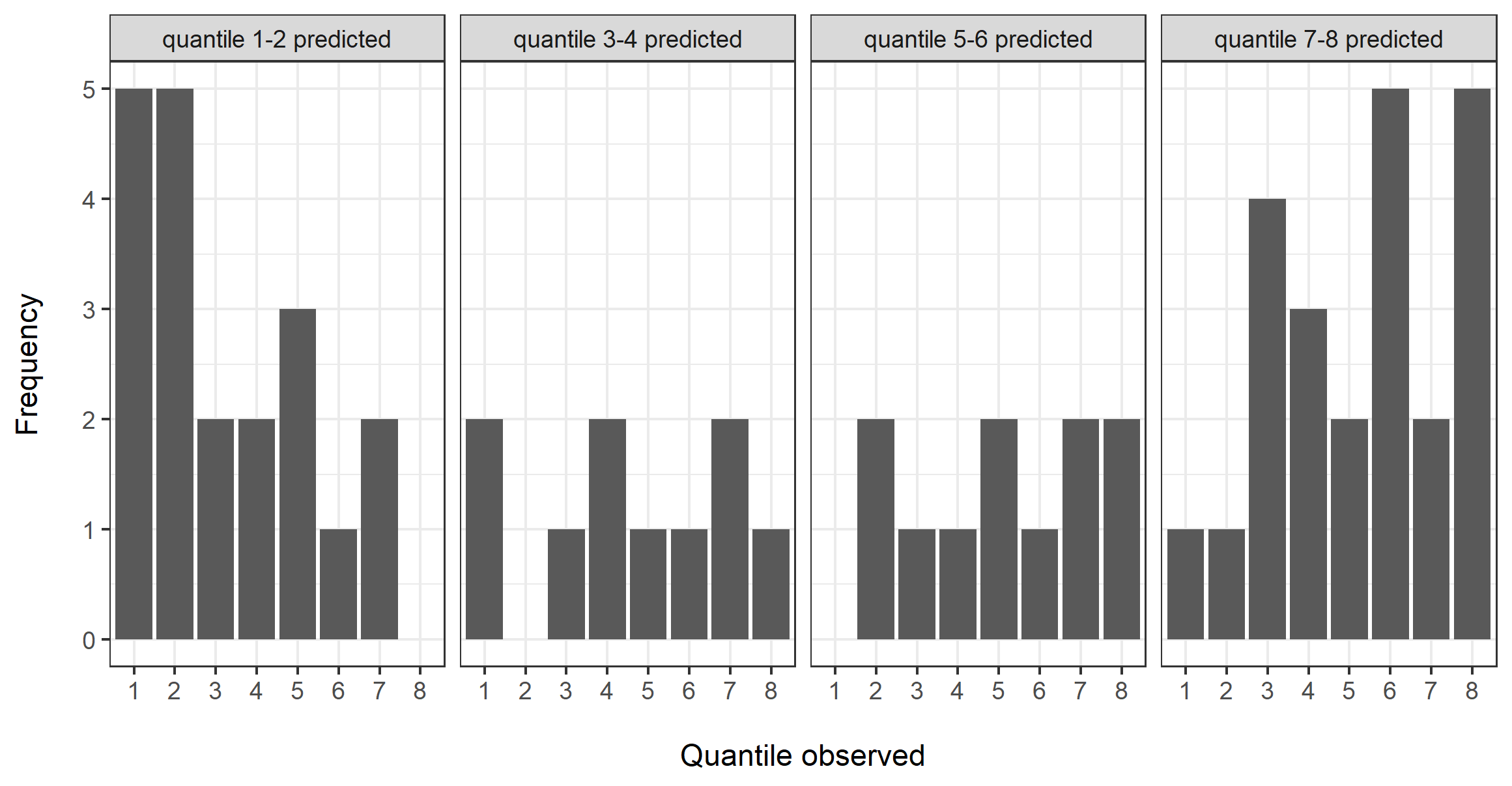


**Figure S5.** Common support for estimating the effect of earlier syntax input $Z_{1S}$(parent clauses per sentence at 14 months).


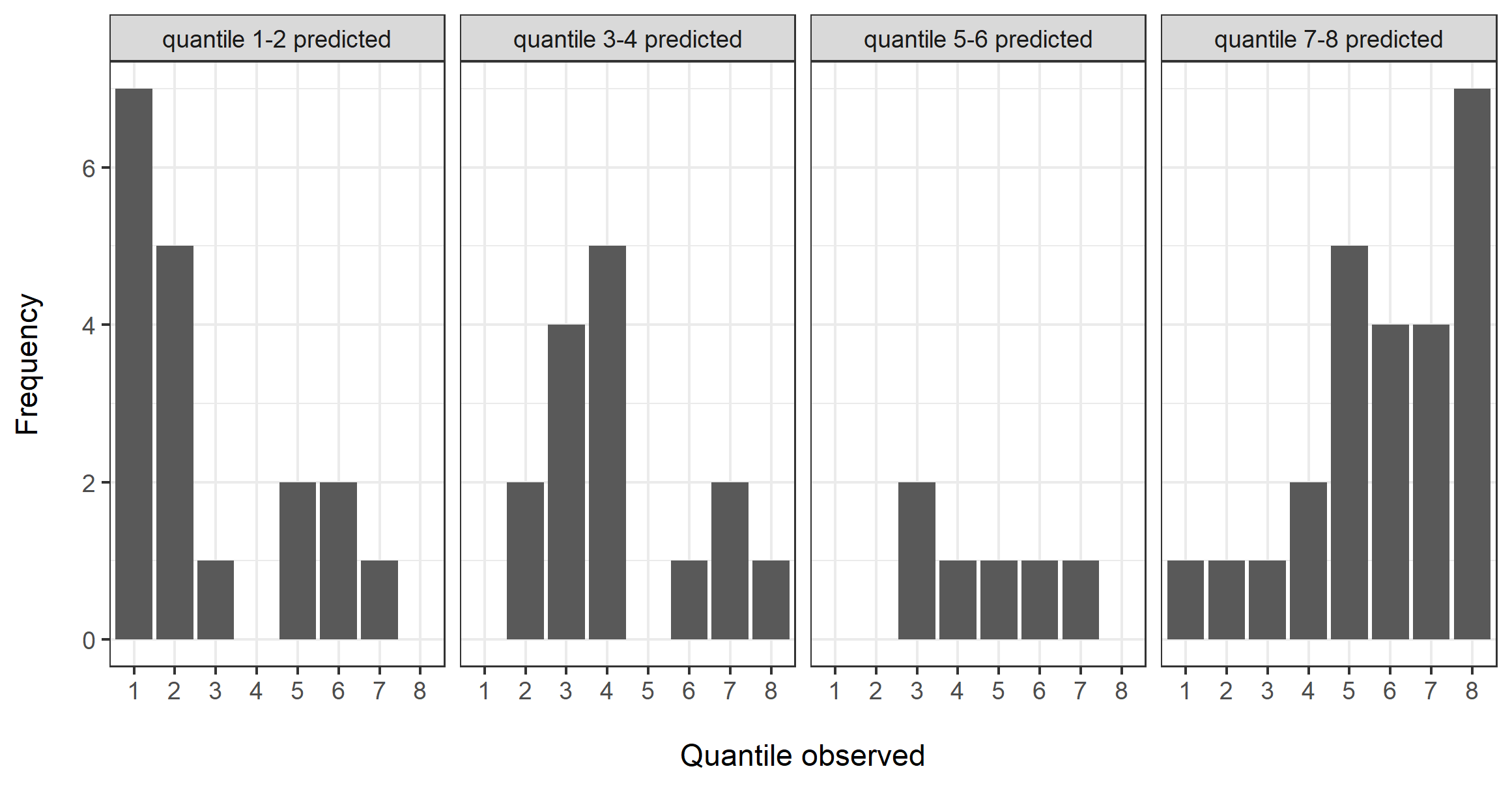


**Figure S6.** Common support for estimating the effect of later syntax input $Z_{2S}$(parent clauses per sentence at 30 months).

**Section S8.** Sensitivity analysis

We assess the sensitivity of our results in two ways. Firstly, we check the level of bias we might expect if there happen to be confounders we have not observed. Secondly, we check the potential impact of our models being mis-specified in terms of functional form.

For assessing the potential biasing effect of unobserved confounders on our estimates, we took the following approach. The most important confounders for parent and child language reported in previous literature are parent IQ and household income, which we observe. However, weaker confounders that we do not observe may nonetheless exist. How sensitive are the results to the failure to include these potential unobserved covariates? We want to find the range of possible biases stemming from the presence of unobserved covariates whose confounding effect is similar to those of the covariates we observed, once the effects of parent IQ and household income are accounted for. If it is the case that controlling for IQ and income alone is nearly sufficient to remove confounding even of other variables that are prime candidates as confounders, then we would regard the results as insensitive. This approach can be regarded as a partial specification check. We emphasize that this is a very partial test of sensitivity and should not be regarded as proof that our results are valid. One simply cannot know the severity of bias resulting from failure to include unobserved covariates.

To perform our sensitivity analysis, we assume our best-fitting model in each case: the constant effects model for vocabulary, and the differing effects model for syntax.

***Vocabulary***

**True Model**

$\begin{matrix} Y_{i}=\delta Z_{i}+\gamma U_{i}+e_{i}, Cov(Z_{i},e_{i})=\sigma_{ze}=0 \\ U_{i}=\alpha Z_{i}+\varepsilon_{i} Cov(Z_{i},\varepsilon_{i})=\sigma_{z\varepsilon}=0 \end{matrix}$ (S3)

Here, $Y_{i}$ = residual of the outcome unexplained by parent IQ and income, $Z_{i}$ = residual of cumulative parent vocabulary input unexplained by parent IQ and income, and $U_{i}$ = the same residual for the unobserved covariate.

**Proposition**: If we regress *Y* on *Z*, omitting *U*, we identify the “prima facie” regression coefficient $\delta_{PF}\equiv\sigma_{zy}/\sigma_{z}^{2}=\delta+\gamma\alpha$ where $\gamma\alpha$is the bias.

**Proof:**

$\begin{matrix} Z_{i}Y_{i}=\delta Z_{i}^{2}+\gamma Z_{i}(\alpha Z_{i}+\varepsilon_{i})+Z_{i}e \\ =Z_{i}^{2}(\delta+\gamma\alpha)+\gamma Z_{i}\varepsilon_{i}+e_{i}Z \end{matrix}$ (S4)

Now take expectations:

$$\begin{matrix} E(Z_{i}Y_{i})=\sigma_{zy}=E(Z_{i}^{2})(\delta+\gamma\alpha)+\gamma E(Z_{i}\varepsilon_{i})+E(e_{i}Z) \\ =\sigma_{z}^{2}(\delta+\gamma\alpha)+\gamma\sigma_{z\varepsilon}+\sigma_{ze} \end{matrix}$$

Now, assuming $\sigma_{z\varepsilon}=\sigma_{ze}=0$and dividing by $\sigma_{z}^{2}$, we have

$\sigma_{zy}/\sigma_{z}^{2}\equiv\delta_{PF}=\delta+\gamma\alpha$ (S5)

Now we need to derive $\gamma,\alpha$.

The second equation in (S1) is a simple linear regression, therefore,

$\alpha=\sigma_{zu}/\sigma_{z}^{2}=\rho_{zu}\sigma_{u}/\sigma_{z}$ (S6)

From the first equation in (S1), we see that

$\begin{matrix} \left(\begin{matrix} \gamma\\ \delta\end{matrix}\right)=\left(\begin{matrix} \sigma_{u}^{2} & \sigma_{uz} \\ \sigma_{uz} & \sigma_{z}^{2} \end{matrix}\right)^{-1}\left(\begin{matrix} \sigma_{uy} \\ \sigma_{zy} \end{matrix}\right) \\ \equiv\left(\begin{matrix} \sigma^{uu} & \sigma^{uz} \\ \sigma^{uz} & \sigma^{zz} \end{matrix}\right)\left(\begin{matrix} \sigma_{uy} \\ \sigma_{zy} \end{matrix}\right) \end{matrix}$ (S7)

where

$\begin{matrix} \sigma^{uu}=\left(\sigma_{u}^{2}-\frac{\sigma_{uz}^{2}}{\sigma_{z}^{2}}\right)^{-1}=\left[\sigma_{u}^{2}\left(1-\frac{\sigma_{uz}^{2}}{\sigma_{u}^{2}\sigma_{z}^{2}}\right)\right]^{-1}=\frac{1}{\sigma_{u}^{2}(1-\rho_{uz}^{2})} \\ \sigma^{uz}=-\sigma^{uu}\frac{\sigma_{uz}}{\sigma_{z}^{2}}. \end{matrix}$ (S8)

Therefore,

$$\begin{matrix} \gamma=\frac{\sigma_{uy}-\sigma_{uz}\sigma_{zy}/\sigma_{z}^{2}}{\sigma_{u}^{2}(1-\rho_{uz}^{2})}=\frac{\sigma_{uy}/\sigma_{u}^{2}-(\sigma_{uz}/\sigma_{u}^{2})(\sigma_{zy}/\sigma_{z}^{2})}{(1-\rho_{uz}^{2})} \\ =\frac{\beta_{y|u}-\beta_{z|u}\beta_{y|z}}{1-\rho_{uz}^{2}}=\frac{\rho_{uy}\frac{\sigma_{y}}{\sigma_{u}}-\rho_{zu}\frac{\sigma_{z}}{\sigma_{u}}\rho_{yz}\frac{\sigma_{y}}{\sigma_{z}}}{1-\rho_{uz}^{2}} \\ =\frac{(\rho_{uy}-\rho_{zu}\rho_{yz})\frac{\sigma_{y}}{\sigma_{u}}}{1-\rho_{uz}^{2}} \end{matrix}$$

Thus, the components of the bias in the vocabulary case are:

$\alpha=\sigma_{zu}/\sigma_{z}^{2}=\rho_{zu}\sigma_{u}/\sigma_{z}$ (S9)

$\gamma=\frac{(\rho_{uy}-\rho_{zu}\rho_{yz})\frac{\sigma_{y}}{\sigma_{u}}}{1-\rho_{uz}^{2}}$ (S10)

To calculate the bias, we treat each of our observed covariates in turn as if they were unobserved, and assess the difference between the prima facie estimate we would obtain if this covariate were omitted and the estimate we would obtain from the “corrected model” if it were included.

The table below shows the results of the sensitivity analysis for vocabulary. The discrepancies between each “corrected model” and the flawed model that accounted only for IQ and income were negligible except in the case of child gesture. Failure to include an unobserved covariate with a confounding effect similar to child gesture would exaggerate the estimate of the effect of vocabulary input by about 24%. Even this discrepancy, however, would not incline us to draw qualitatively incorrect conclusions.

| **Potential *u*** | **Estimated bias** | $\delta_{V}$ **estimate**  **corrected for bias** |
| --- | --- | --- |
| Child birth order | -0.00020 | 0.02075 |
| Gender | 0.00052 | 0.02003 |
| Child word types at 14 months | 0.00185 | 0.01870 |
| Child gesture types at 14 months | 0.00400 | 0.01654 |
| Parent education | 0.00092 | 0.01962 |

***Syntax***

**True Model**

$\begin{matrix} Y_{i}=\delta_{1}Z_{1i}+\delta_{2}Z_{2i}+\gamma U_{i}+e \\ U_{i}=\alpha_{1}Z_{i}+\alpha_{2}Z_{2i}+\varepsilon_{i} \end{matrix}$ (S11)

Here, $Y_{i}$ = residual of the outcome unexplained by parent IQ and income, = residual of earlier parental syntax input unexplained by parent IQ and income, = residual of later parental syntax input unexplained by parent IQ and income, and $U_{i}$ = the same residual for the unobserved covariate.

Rewrite in matrix notation:

$\begin{matrix} Y_{i}=\left(\begin{matrix} Z_{1i} & Z_{2i} \end{matrix}\right)\left(\begin{matrix} \delta_{1} \\ \delta_{2} \end{matrix}\right)+\gamma U_{i}+e\equiv Z_{i}^{T}\delta+\gamma U_{i}+e_{i} \\ U_{i}=\left(\begin{matrix} Z_{1i} & Z_{2i} \end{matrix}\right)\left(\begin{matrix} \alpha_{1} \\ \alpha_{2} \end{matrix}\right)+\varepsilon=Z_{i}^{T}\alpha+\varepsilon_{i} \end{matrix}$ (S12)

where

$Z_{i}=\left(\begin{matrix} Z_{1i} \\ Z_{2i} \end{matrix}\right),\delta=\left(\begin{matrix} \delta_{1} \\ \delta_{2} \end{matrix}\right), \alpha=\left(\begin{matrix} \alpha_{1} \\ \alpha_{2} \end{matrix}\right)$ (S13)

Derivation of Bias:

$\begin{matrix} Z_{i}Y_{i}=Z_{i}Z_{i}^{T}\delta+\gamma Z_{i}U_{i}+Z_{i}e_{i} \\ =Z_{i}Z_{i}^{T}\delta+\gamma Z_{i}(Z_{i}^{T}\alpha+\varepsilon)+Z_{i}e_{i} \\ =Z_{i}Z_{i}^{T}\delta+\gamma Z_{i}Z_{i}^{T}\alpha+\gamma Z_{i}\varepsilon+Z_{i}e_{i} \end{matrix}$ (S14)

Now take expectations:

$\begin{matrix} E(Z_{i}Y_{i})=E(Z_{i}Z_{i}^{T})\delta+\gamma E(Z_{i}Z_{i}^{T})\alpha+\gamma E(Z_{i}\varepsilon)+E(Z_{i}e_{i}) \\ =\Sigma_{zy}=\Sigma_{zz}\delta+\gamma\Sigma_{zz}\alpha+\gamma\Sigma_{z\varepsilon}+\Sigma_{ze} \end{matrix}$ (S15)

Therefore, given that $\Sigma_{z\varepsilon}=\Sigma_{ze}=0$,

$\Sigma_{zz}^{-1}\Sigma_{zy}=\delta_{PF}=\delta+\gamma\alpha$ (S16)

Now we need to derive$\gamma,\alpha$. If we assume a missing covariate is associated with each *Z* and with *Y*, the results become very complex and uninterpretable. Instead, we consider the sensitivity of our estimates of $\delta_{1}$(the effect of earlier parent syntax input) and $\delta_{2}$(the effect of later parent syntax input) separately. That is, we assume either the model $U_{i}=\alpha_{1}Z_{1i}+\varepsilon_{i}$ and use this to study sensitivity regarding our inference about $\delta_{1}$or we assume $U_{i}=\alpha_{2}Z_{2i}+\varepsilon_{i}$in order to study sensitivity about our inference regarding$\delta_{2}$. For inferences about each $\delta$, the results are as follows:

The second equation in (1) is a simple linear regression therefore,

$\alpha=\sigma_{zu}/\sigma_{z}^{2}=\rho_{zu}\sigma_{u}/\sigma_{z}$ (S17)

From the first equation in (1), we see that

$\begin{matrix} \left(\begin{matrix} \gamma\\ \delta\end{matrix}\right)=\left(\begin{matrix} \sigma_{u}^{2} & \sigma_{uz} \\ \sigma_{uz} & \sigma_{z}^{2} \end{matrix}\right)^{-1}\left(\begin{matrix} \sigma_{uy} \\ \sigma_{zy} \end{matrix}\right) \\ \equiv\left(\begin{matrix} \sigma^{uu} & \sigma^{uz} \\ \sigma^{uz} & \sigma^{zz} \end{matrix}\right)\left(\begin{matrix} \sigma_{uy} \\ \sigma_{zy} \end{matrix}\right) \end{matrix}$ (S18)

where

$\begin{matrix} \sigma^{uu}=\left(\sigma_{u}^{2}-\frac{\sigma_{uz}^{2}}{\sigma_{z}^{2}}\right)^{-1}=\left[\sigma_{u}^{2}\left(1-\frac{\sigma_{uz}^{2}}{\sigma_{u}^{2}\sigma_{z}^{2}}\right)\right]^{-1}=\frac{1}{\sigma_{u}^{2}(1-\rho_{uz}^{2})} \\ \sigma^{uz}=-\sigma^{uu}\frac{\sigma_{uz}}{\sigma_{z}^{2}}. \end{matrix}$ (S19)

Therefore,

$$\begin{matrix} \gamma=\frac{\sigma_{uy}-\sigma_{uz}\sigma_{zy}/\sigma_{z}^{2}}{\sigma_{u}^{2}(1-\rho_{uz}^{2})}=\frac{\sigma_{uy}/\sigma_{u}^{2}-(\sigma_{uz}/\sigma_{u}^{2})(\sigma_{zy}/\sigma_{z}^{2})}{(1-\rho_{uz}^{2})} \\ =\frac{\beta_{y|u}-\beta_{z|u}\beta_{y|z}}{1-\rho_{uz}^{2}}=\frac{\rho_{uy}\frac{\sigma_{y}}{\sigma_{u}}-\rho_{zu}\frac{\sigma_{z}}{\sigma_{u}}\rho_{yz}\frac{\sigma_{y}}{\sigma_{z}}}{1-\rho_{uz}^{2}} \\ =\frac{(\rho_{uy}-\rho_{zu}\rho_{yz})\frac{\sigma_{y}}{\sigma_{u}}}{1-\rho_{uz}^{2}} \end{matrix}$$

The components of the bias for $\delta_{1}$ in the syntax case are therefore:

$\alpha_{1}=\sigma_{z_{1}u}/\sigma_{z_{1}}^{2}=\rho_{z_{1}u}\sigma_{u}/\sigma_{z_{1}}$ (S20)

$\gamma=\frac{[(1-\rho_{12}^{2})\rho_{uy}-\rho_{1u}\rho_{y1}-\rho_{y2}\rho_{1u}\rho_{21}]\frac{\sigma_{y}}{\sigma_{u}}}{1-\rho_{12}^{2}-\rho_{1u}^{2}}$ (S21)

where $\rho_{12}=Corr(Z_{1i},Z_{2i}), \rho_{1u}=Corr(Z_{1i},U_{i}), \rho_{y1}=Corr(Y_{i},Z_{1i}), \rho_{y2}=Corr(Y_{i},Z_{2i}).$

We performed a sensitivity analysis assessing the potential biasing effect of unobserved confounders on our estimates of the effects of earlier and later syntax input. The results are reported in the tables below. For $\delta_{1}$, our estimate of the effect of earlier syntax input, we find that ignoring our worst “unobserved” confounder would exaggerate our estimate of the negative effect of earlier input by around 18%. For $\delta_{2}$, our estimate of the effect of later syntax input, ignoring our worst “unobserved” confounder would exaggerate our estimate of the positive effect of later input by around 9%. As for the vocabulary analysis, our conclusions would not qualitatively change in either case.

| **Potential *u*** | **Estimated bias** | $\delta_{1S}$ **estimate**  **corrected for bias** |
| --- | --- | --- |
| Child birth order | -0.03292 | -0.20321 |
| Gender | -0.00967 | -0.22646 |
| Child word types at 14 months | -0.03517 | -0.20096 |
| Child gesture types at 14 months | -0.02635 | -0.20977 |
| Parent education | -0.00224 | -0.23389 |

| **Potential *u*** | **Estimated bias** | $\delta_{2S}$ **estimate**  **corrected for bias** |
| --- | --- | --- |
| Child birth order | 0.00348 | 0.21245 |
| Gender | 0.00271 | 0.21322 |
| Child word types at 14 months | 0.01167 | 0.20426 |
| Child gesture types at 14 months | 0.01547 | 0.20045 |
| Parent education | 0.01835 | 0.19757 |

We also assess the robustness of our models to violations of the assumption that the relationship between input and outcome is linear. To do this, we compare the outcomes predicted by our best-fitting linear models to the outcomes predicted when assuming a quadratic or logarithmic relationship between input and outcome. Figure S7 below compares predicted outcomes for linear, logarithmic, and quadratic models over the range of observed input between the 10^th^ and 90^th^ percentiles for (a) cumulative vocabulary input, (b) earlier syntax input, and (c) later syntax input. The relationship between input and outcome appears similar across the three models, suggesting that our findings are robust to choice of functional form.

a)


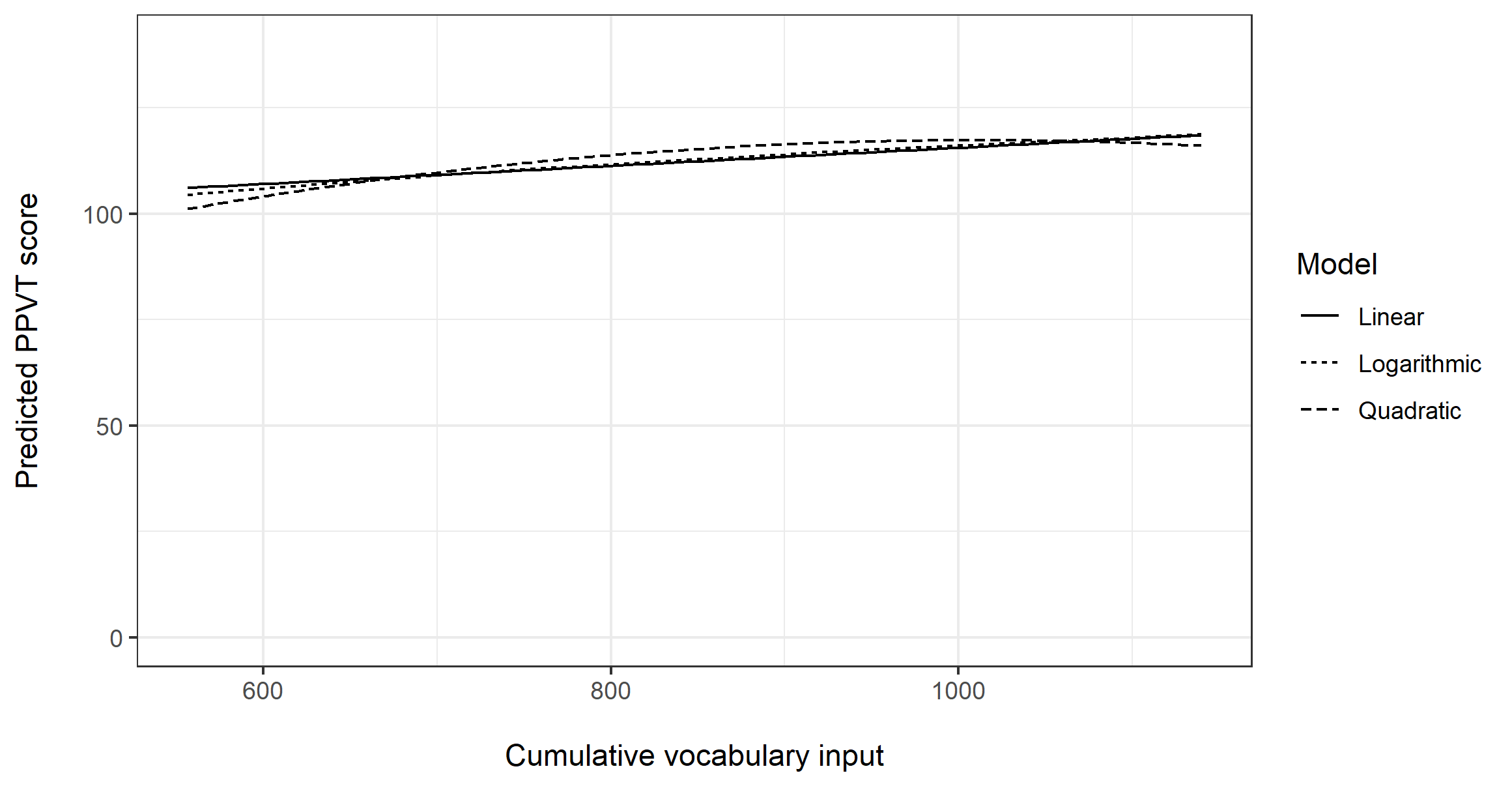


b)


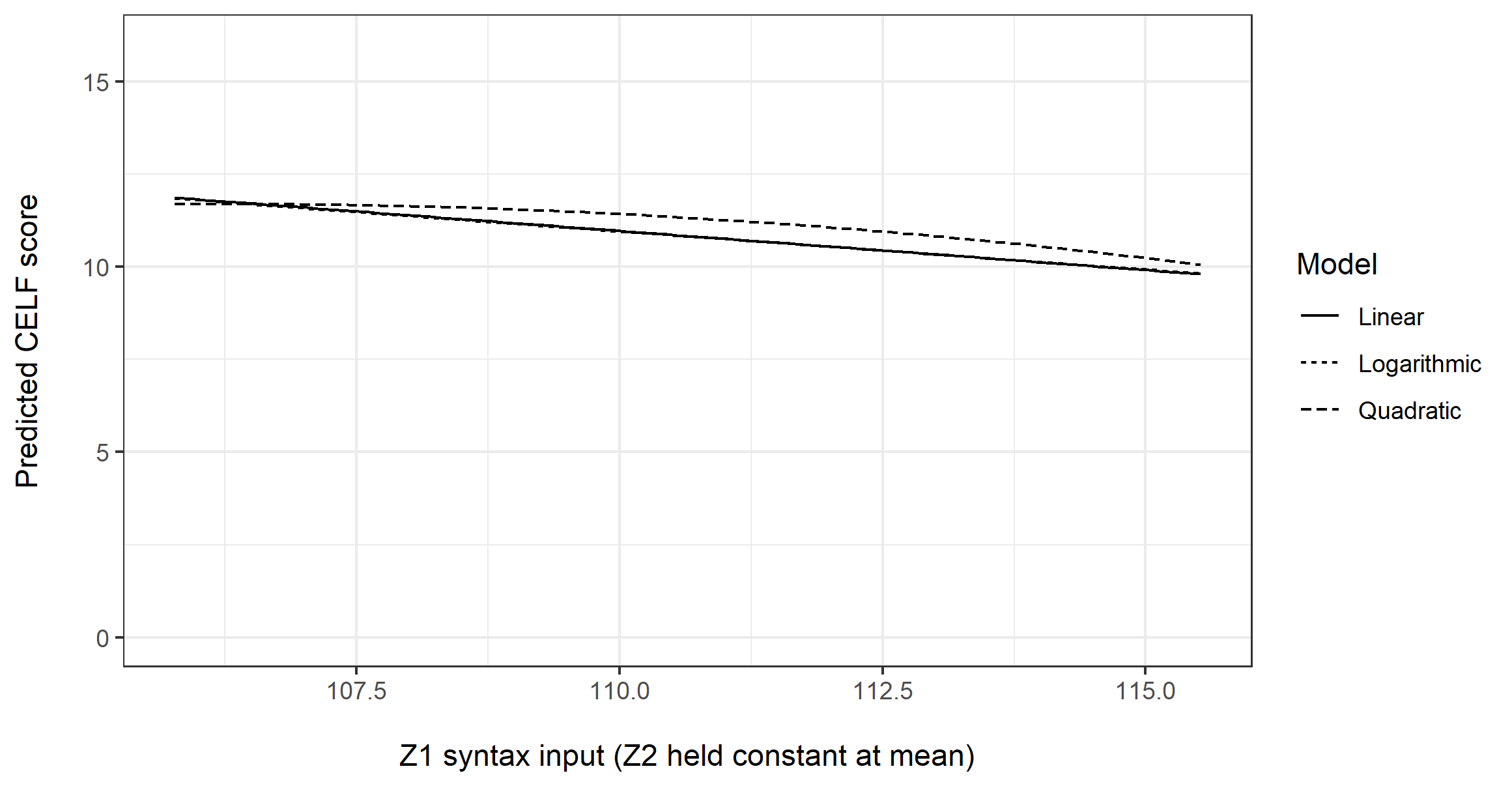


c)


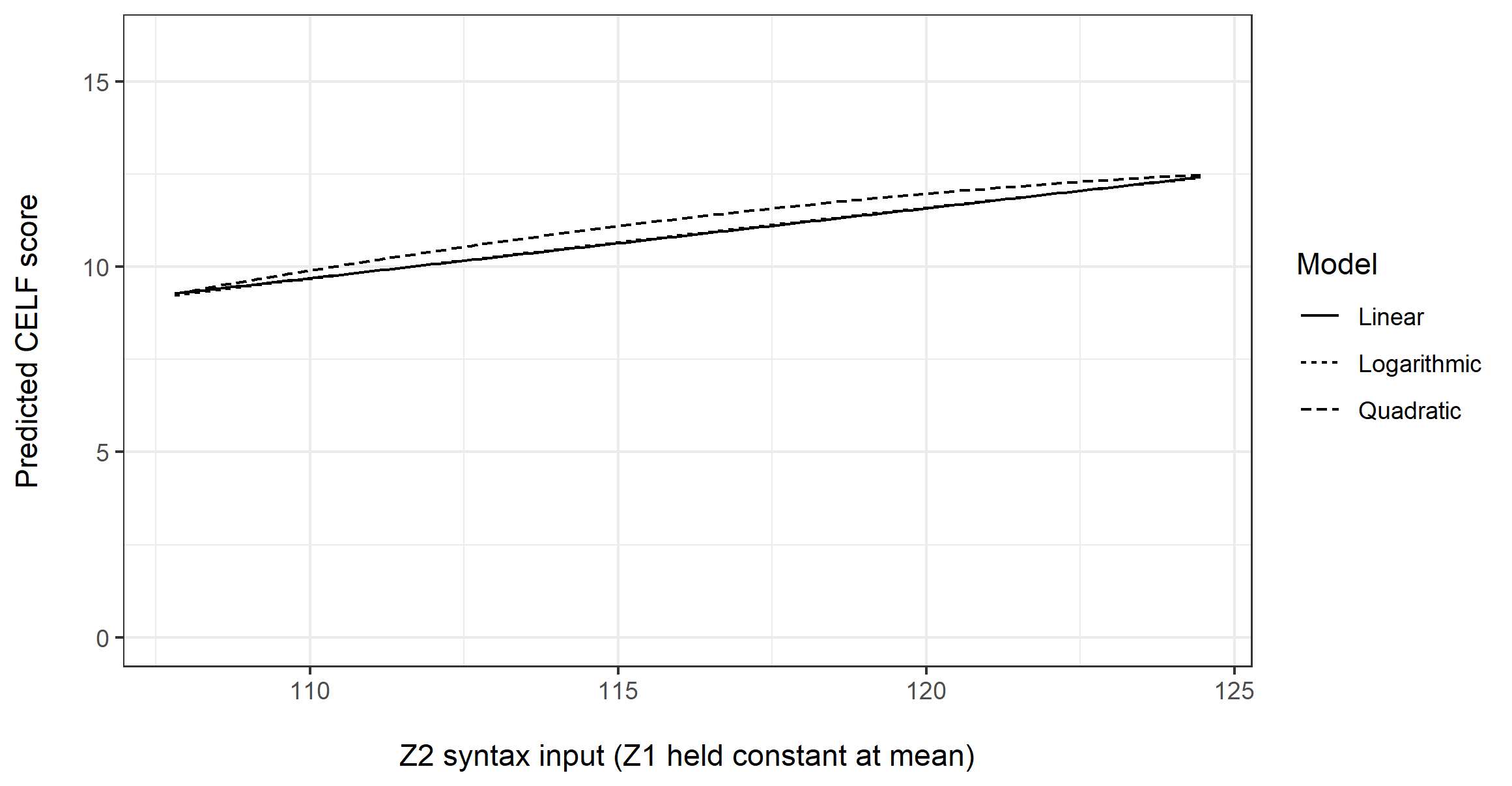


**Figure S7.** Predicted outcomes (y axis) for different levels of input (x axis) according to our best models for a) vocabulary, b) early syntax, and c) later syntax. Separate lines show outcomes predicted where the relationship between input and outcome is modeled as linear, logarithmic, and quadratic.

**Supplemental References**

van Buuren, S. (2018). *Flexible imputation of missing data*. London: Chapman and Hall/CRC.

van Buuren, S., & Groothuis-Oudshoorn, K. (2011). mice: Multivariate Imputation by Chained Equations in R. *Journal of Statistical Software*, *45*, 1-67.

Gardner, H., Froud, K., McClelland, A., & van der Lely, H. K. (2006). Development of the Grammar and Phonology Screening (GAPS) test to assess key markers of specific language and literacy difficulties in young children. *International Journal of Language & Communication Disorders*, *41*(5), 513-540.

Hong, G. (2012). Marginal mean weighting through stratification: a generalized method for evaluating multivalued and multiple treatments with nonexperimental data. *Psychological Methods*, *17*(1), 44–60.

Little, R. J. A. (1988). Missing-data adjustments in large surveys. *Journal of Business & Economic Statistics*, *6*, 287-296.

Naimi, A. I., Moodie, E. E. M., Auger, N., & Kaufman, J. S. (2014). Constructing inverse probability weights for continuous exposures: A comparison of methods. *Epidemiology*, *25*(2), 292–299.

Raudenbush, S. W., & Bryk, A. S. (2002). *Hierarchical linear models: Applications and data analysis methods*. Thousand Oaks: SAGE Publishing.

Rubin, D. B. (1986). Statistical matching using file concatenation with adjusted weights and multiple imputations. *Journal of Business & Economic Statistics*, *4*, 87-94.

Rubin, D. B. (1987). *Multiple imputation for nonresponse in surveys*. London: John Wiley & Sons.
